# Engineered molecular sensors for quantifying cell surface crowding

**DOI:** 10.1073/pnas.2219778120

**Published:** 2023-05-15

**Authors:** Sho C. Takatori, Sungmin Son, Daniel S. W. Lee, Daniel A. Fletcher

**Affiliations:** ^a^Department of Bioengineering, University of California, Berkeley, CA 94720; ^b^Department of Chemical Engineering, University of California, Santa Barbara, CA 93106; ^c^Department of Bio and Brain Engineering, Korea Advanced Institute of Science and Technology, Daejeon, Republic of Korea; ^d^University of California, Berkeley/University of California, San Francisco Graduate Group in Bioengineering, Berkeley, CA 94720; ^e^Division of Biological Systems and Engineering, Lawrence Berkeley National Laboratory, Berkeley, CA 94720; ^f^Chan Zuckerberg Biohub, San Francisco, CA 94158

**Keywords:** cell surface crowding, cancer cell biology, glycocalyx, biophysics, plasma membrane

## Abstract

Cells mediate interactions with the extracellular environment through a crowded assembly of transmembrane proteins, glycoproteins and glycolipids on their plasma membrane. The extent to which surface crowding modulates the biophysical interactions of ligands, receptors, and other macromolecules is poorly understood due to the lack of methods to quantify surface crowding on native cell membranes. In this work, we demonstrate that physical crowding on reconstituted membranes and live cell surfaces attenuates the effective binding affinity of macromolecules such as IgG antibodies in a surface crowding-dependent manner. We combine experiment and simulation to design a crowding sensor based on this principle that provides a quantitative readout of cell surface crowding. Our measurements reveal that surface crowding decreases IgG antibody binding by 2 to 20 fold in live cells compared to a bare membrane surface. Our sensors show that sialic acid, a negatively charged monosaccharide, contributes disproportionately to red blood cell surface crowding via electrostatic repulsion, despite occupying only ~1% of the total cell membrane by mass. We also observe significant differences in surface crowding for different cell types and find that expression of single oncogenes can both increase and decrease crowding, suggesting that surface crowding may be an indicator of both cell type and state. Our high-throughput, single-cell measurement of cell surface crowding may be combined with functional assays to enable further biophysical dissection of the cell surfaceome.

## Significance

Cells interact with each other and the extracellular environment through a crowded assembly of polymers on their plasma membranes. The high density of these surface polymers can generate physical crowding that impacts cell function. However, tools to quantify the extent and effect of surface crowding on live cell membranes are lacking. In this work, we design macromolecular sensors that act as reporters of cell surface crowding. We combine experiments on reconstituted and live cell surfaces with molecular dynamics simulations to provide a mechanistic understanding of how cell surface crowding reduces binding of soluble molecules, and we show that crowding varies significantly with cell type and is affected by oncogene expression.

The biophysical organization of proteins, glycoproteins, and glycolipids that densely coat the surface of the cell membrane has been shown to govern many important physiological processes. Physical crowding on cell surfaces has a connection to cancer malignancy (1–3), and the dense glycocalyx on cancer cell surfaces can sterically hinder antibody binding and phagocytosis by immune cells (4). Recent studies have shown that the glycocalyx can also attenuate the binding of viruses and lectins to cell surface receptors (5–7). Surface crowding also alters protein mobility and sorting (8), as well as membrane channel gating (9). However, quantitative methods to obtain a detailed, mechanistic understanding of plasma membrane density and the biophysical interactions that govern macromolecular binding on live cell surfaces are lacking. This leaves basic questions about cell surface crowding unanswered, including the extent to which glycosylation contributes, how crowding differs among cell types and states, and even how best to quantify crowding in live cells.

While proteomic analysis of cell surface proteins provide detailed information on the relative abundance of proteins at the population level (10), it is difficult to predict collective biophysical features of cell surfaces simply from knowledge of the surface proteome. Previous studies have shown that the physical accessibility of large soluble ligands and macromolecules decreases on synthetic surfaces grafted with synthetic polymers or purified proteins (11–14). Other studies have developed tools that measure effects of surface crowding, including Houser et al. (15), who measured the separation of Förster resonance energy transfer pairs as a function of the steric interactions within the surface polymers on reconstituted membranes, and Son et al. (16), who measured nanometer-scale changes in height of multidomain proteins in vitro as surface density increased. While these studies of reconstituted systems provide valuable insights, direct quantification of surface crowding on live cell membranes remains a challenge. Advanced imaging techniques like electron microscopy enable nanometer-scale visualization of the cell membrane (17), but the preparation process is destructive, and structural information does not easily translate to a physical understanding of the effects of crowding, leaving a need for new tools to study the surfaces of living cells (3).

Here, we report a simple approach to measure surface crowding on live cells. Inspired by theoretical work on the adsorption of macromolecules on crowded surfaces (18), we engineered macromolecular probes that insert into bilayer membranes and quantify the repulsive penalty posed by crowded cell surfaces by a reduction in effective affinity. We first validated the measurement principle by engineering polymer–cholesterol conjugates of varying sizes and measuring their effective binding affinity on reconstituted membranes and red blood cell (RBC) membranes. We quantified the reduction in binding affinity as a crowding energy using molecular dynamics (MD) simulations and adsorption theories. We then engineered a two-component sensor based on the binding of a monoclonal anti-biotin antibody to a biotin–fluorophore–cholesterol conjugate that inserts into live cell plasma membranes. By measuring the fraction of antibody bound to the surface biotin on individual cells relative to bare model membranes, we quantify the effect of cell surface crowding on antibody-binding affinity. Our crowding sensors work on cells of different size, shape, and membrane lipid compositions, providing a technique to compare surface crowding across different live cell types and states.

## Results

### Physical Crowding on Membrane Surfaces Reduces the Binding Affinity of Soluble Macromolecules.

**Fig. 1.**
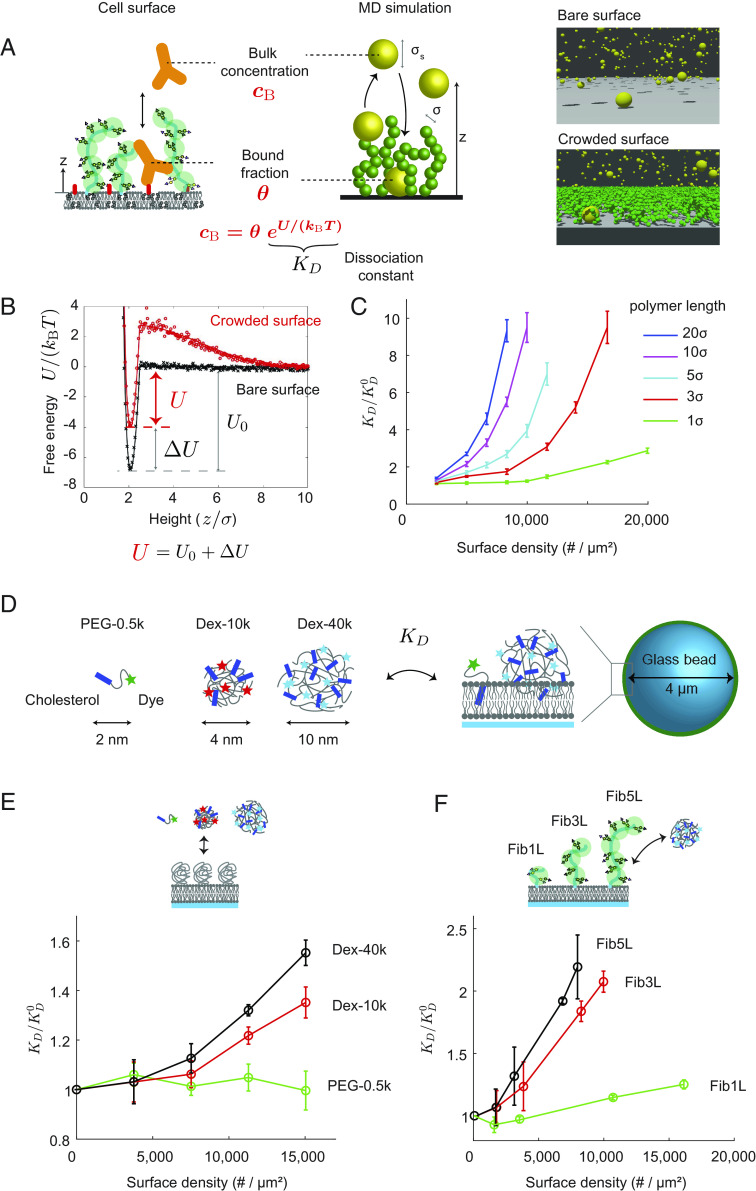
Macromolecular binding is a reporter of surface crowding. (*A*) Soluble macromolecules, like monoclonal antibodies, experience a repulsive energy penalty when binding to antigen targets located on crowded surfaces. Therefore, the concentration of surface-bound macromolecules is a direct readout of the effective dissociation constant and the crowding state of the surface. (*Right*) Snapshots of coarse-grained molecular dynamics (MD) simulations on bare and crowded surfaces. Soluble macromolecules (yellow spheres) of size $\sigma_{s}$ bind onto a surface functionalized with multidomain protein polymers (green spheres) with density, n , and monomer size, σ . (*B*) MD simulations are used to calculate the binding energy of soluble macromolecules on bare (black symbols) and crowded (red symbols) surfaces. The binding affinity of soluble macromolecules on crowded surfaces ( U ) is smaller than that on bare surfaces ( $U_{0}$ ). The difference, $\Delta U=U-U_{0}$ , is related to the crowding-induced change in the effective dissociation constant, $K_{D}$ . Polymer brush theories are used to relate the effective dissociation constant to surface crowding (solid curves). See *SI Appendix* for further theoretical and computational results. (*C*) The dissociation constant, $K_{D}$ , normalized by the bare-surface value, $K_{D}^{0}$ , increases monotonically with the surface protein density and contour length. (*D*) Experimental design of cholesterol-based sensors to measure crowding on membranes. Synthetic polymers of different molecular weights are used to vary the overall size of the sensor. The sensors have a strong binding affinity to the lipid bilayer, and fluorescent labels provide a readout of bound surface concentration and the effective dissociation constant, $K_{D}$ . (*E*) Normalized dissociation constant, $K_{D}/K_{D}^{0}$ , of the small (PEG-0.5k), medium (Dex-10k), and large (Dex-40k) sensors increases on lipid-coated beads functionalized with PEG-3k at varying surface densities. $K_{D}^{0}$ is the affinity on bare lipid-coated beads. (*F*) $K_{D}/K_{D}^{0}$ of large Dex-40k sensors increases on lipid-coated beads functionalized with engineered proteins of the FNIII domain repeats, Fibcon (Fib), which has a size of ~4 nm per domain. Fib1L, Fib3L, and Fib5L contain 1, 3, and 5 domains, respectively. For all data, error bars indicate SDs of the mean; N > 3.

Existing theories of adsorption thermodynamics give a direct relationship between the dissociation constant of a soluble macromolecule and the free energy of the surface, $K_{D}=\exp(U/{(k}_{B}T))$ (Fig. 1*A*). Therefore, a measurement of the $K_{D}$ on a surface is a reporter of its free energy. By measuring the dissociation constants on a crowded surface, $K_{D}$ , and a bare surface, $K_{D}^{0}$ , we can measure the energy penalty posed by surface crowding, $\Delta U=k_{B}T\;ln\;\left(K_{D}/K_{D}^{0}\right)$ . To test whether we could use this principle to read out surface crowding, we conducted coarse-grained MD simulations to study the binding of macromolecules to a bare surface and to a surface decorated with polymers (Fig. 1*A*). We found that the free energy as a function of distance from the surface increases in the presence of surface-tethered polymers (Fig. 1*B*), and the deviation of the energy minima is a direct readout of the effective dissociation constant, $K_{D}$ (see *SI Appendix* for further detail). We plot all results as a ratio of $K_{D}/K_{D}^{0}$ , since the crowding energy is given by $\Delta U=k_{B}T\;ln\;\left(K_{D}/K_{D}^{0}\right)$ and only relative differences are important for surface crowding. As expected, we find that $K_{D}/K_{D}^{0}$ increases as we increase the surface crowding by changing either the surface density or the contour length of the crowding polymers (Fig. 1*C*). It is important to note that the contour length of the surface polymers has a strong effect on $K_{D}$ , which demonstrates that the polymer number density alone is an insufficient metric of surface crowding.

Guided by these predictions, we engineered a series of exogenous macromolecular probes with known size and affinity to a lipid bilayer by conjugating cholesterol and fluorescent dyes to PEG 1k, dextran 10k, and dextran 40k macromolecules (Fig. 1*D* and *Materials and Methods*). The diameters of these probes are approximately 2, 4, and 10 nm based on the radii of gyration (19). We hypothesized that the reduced binding affinities of these “crowding sensors” to crowded membrane surfaces would act as a reporter of the energy penalty posed by surface crowding. We first tested the binding of our sensors to a supported lipid bilayer (SLB) formed on a glass bead and decorated it with PEG3k polymers simulating cell surface crowding. After allowing the sensor binding to reach equilibrium (45 min), we quantified the bead fluorescence with a flow cytometer. Using an adsorption isotherm to relate the bound sensor concentration to the bulk concentration, we calculated the effective dissociation constant. We found that the $K_{D}$ of the larger dextran 10k and 40k sensors increased on the PEG3k surface compared to that on the bare surface, with the $K_{D}$ of the dextran 40k sensor increasing by 55% when the SLB contained 1% (mol/mol) PEG3k (~15,000/µm^2^ area density) (Fig. 1*E*), which agrees with our simulation and is consistent with previous studies (12). In contrast, the small PEG 0.5k sensor experienced no change in $K_{D}$ for the PEG3k surface densities we tested (Fig. 1*E*). We observed no systematic change in the time to reach equilibrium on crowded surfaces compared to bare surfaces (*SI Appendix*).

To test the sensors’ ability to read out crowding due to proteins rather than synthetic PEG polymers, we then reconstituted SLBs with engineered proteins based on repeats of the FNIII domain, Fibcon, which has a size of ~4 nm per domain (20). We used different lengths based on one (Fib1L), three (Fib3L), or five (Fib5L) domain proteins with decahistidine tags that bind to SLBs containing 1,2-dioleoyl-sn-glycero-3-(N-(5-amino-1-carboxypentyl)iminodiacetic acid)succinyl (DGS-Ni-NTA). The $K_{D}$ of the dextran 40k sensor increased by more than 2× on surfaces crowded with Fib3L and Fib5L at a density of ~10,000/µm^2^ relative to a bare membrane, whereas the $K_{D}$ increased by only ~20% for Fib1L (Fig. 1*F*). It is important to note that the molecular weight of Fib1L (~14 kDa) is 4.6 times larger than PEG3k, yet the crowding strength generated by the PEG surface is 25% larger when comparing them at the same number density. These results highlight the fact that the molecular weight of a surface species is not a proper metric of crowding, just as number density and height of surface species are insufficient metrics by themselves. Indeed, “crowding” as it affects the affinity of soluble molecules at the cell surface is a collective phenomenon that includes these and other cell surface molecular properties.

Our simulations and reconstituted SLB experiments demonstrate the connection between surface crowding and effective binding affinities of large macromolecules. Our simple measurement of $K_{D}$ is a quantitative reporter of surface crowding, regardless of the chemical identity of the surface species. The sensitivity of our sensors to protein length, molecular weight, and density provides a unique approach for studying the biophysical organization of the cell surface.

### Macromolecular Binding at Cell Surfaces Is Osmotically Regulated by Sialylation.

**Fig. 2.**
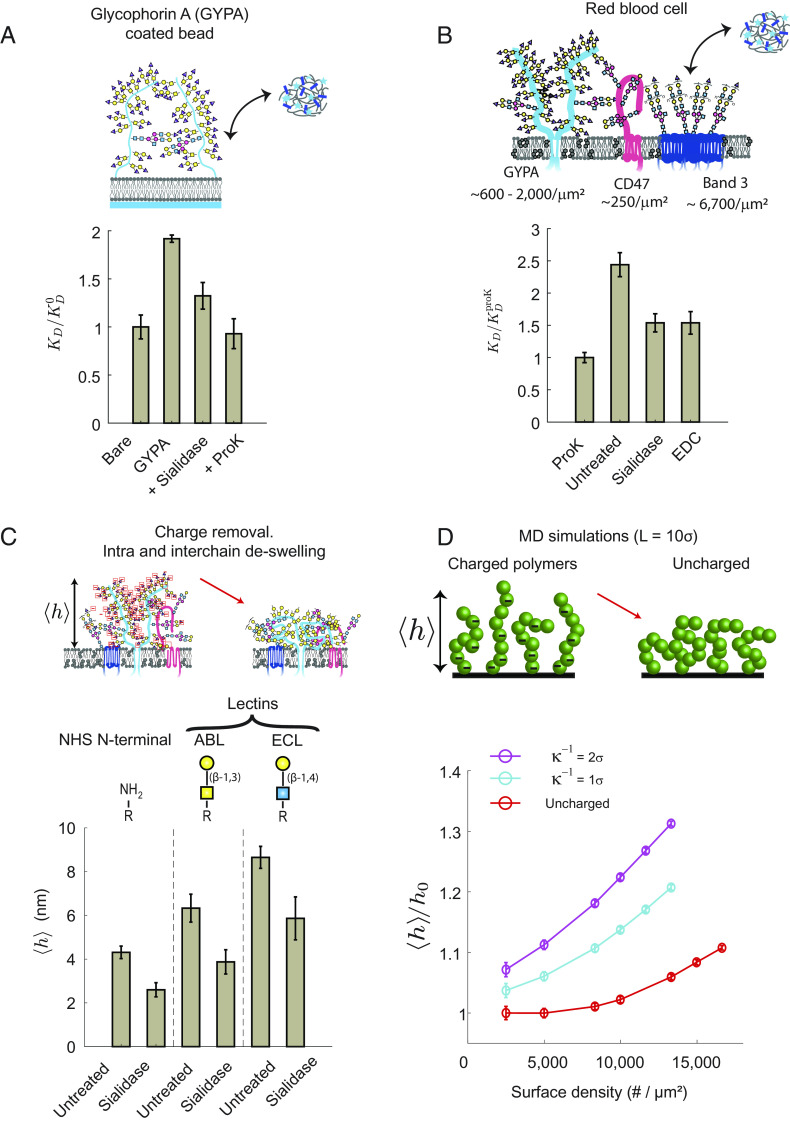
Macromolecular binding on red blood cell (RBC) surfaces is osmotically regulated by sialylation. (*A*) Normalized dissociation constant of the large Dex-40k sensors, $K_{D}/K_{D}^{0}$ , on reconstituted membranes with Glycophorin A (GYPA), a mucin-like glycoprotein found on RBCs. Treatment of the GYPA-bound beads with sialidase removes the sialic acids, which deswells the GYPA and increases the surface accessibility for the sensor. Proteinase K (ProK) removes GYPA and the affinity is restored to the same value as bare beads. (*B*) Dissociation constant of large Dex-40k sensors on RBCs, normalized by the affinity on ProK-treated cells, $K_{D}^{proK}$ . Sialidase treatment removes the sialic acids on the RBC surface and decreases $K_{D}$ , consistent with the results on GYPA-bound beads. EDC carbodiimide chemistry removes the negative charge on the RBC surface. The $K_{D}$ reduction is the same as that with sialidase treatment, consistent with our hypothesis that electrostatic charge repulsion within the glycocalyx reduces the accessibility of the sensors. Note that the affinities are normalized relative to the ProK-treated cells, $K_{D}^{proK}$ , which is different from the baseline on bare lipid-coated beads, $K_{D}^{0}$ . (*C*) Average height of the RBC surface proteins, 〈h〉 , is measured in an untreated cell and cells treated with sialidase using the cell surface optical profilometer (CSOP) (16). The reduction in 〈h〉 with sialidase treatment is consistent with the deswelling of polyelectrolyte brushes from charge removal. Measurements of RBC surface heights are based on N-terminal labeling of proteins, fluorescein 5-isothiocyanate (FITC)-conjugated *Agaricus bisporus* lectin (ABL) targeting the Gal-GalNac disaccharide, FITC-conjugated *Erythrina cristagalli* lectin (ECL) targeting the Gal-GlcNac disaccharide. (*D*) Average normalized height of multidomain proteins in MD simulations with uncharged (red circles) and charged (cyan and magenta circles) protein residues, where $h_{0}$ is the uncharged protein height at dilute densities. Electrostatic interactions among the proteins are modeled by a Yukawa potential with different Debye lengths, $\kappa^{-1}$ . The simulated decrease in surface protein height upon charge removal is consistent with the CSOP data. For all data, error bars indicate SDs of the mean; N > 3.

We next used the crowding sensors to study the effects of glycosylation on crowding on both reconstituted membranes and live plasma membranes. We reconstituted SLBs decorated with purified Glycophorin A (GYPA), a mucin-like transmembrane protein with a heavily glycosylated and sialylated extracellular domain (21). We found that the effective $K_{D}$ of our dextran 40k sensor increased by 2× on the crowded GYPA surface compared to a bare membrane (Fig. 2*A*). When we treated the GYPA-coated beads with sialidase from *Clostridium perfringens* (*Clostridium welchii*), we found that the removal of sialic acid, a negatively charged monosaccharide, decreased $K_{D}$ by 40% compared to the untreated GYPA surfaces. We hypothesized that the negative charge on sialic acids stiffens and stretches the disordered GYPA chain due to intrachain electrostatic repulsion, effectively increasing its persistence length and posing a larger energetic penalty against sensor binding at the surface. Polymer chain stiffening due to electrostatic interactions causes both the osmotic pressure and the effective volume of the polyelectrolyte surface brush to increase, leading to larger $K_{D}$ (see *SI Appendix* for the relationship between $K_{D}$ , osmotic pressure, and glycocalyx volume). GYPA contains 30 sialic acids held in close proximity ( ≈1 nm) in its 15 O-glycans (21), resulting in a large negative charge density along its disordered chain. For end-grafted polymer brushes, strong repulsion among the polymer side chains acts to stiffen the polymers and increase the overall height of the brush (22). We note that interchain interactions would also increase the brush height, but are likely playing a minor role due to screening of charges in physiological buffers. The average spacing between grafting sites of GYPA chains is ≈ 20 nm at 2,000/µm^2^ surface density, which is 30× larger than the 0.7-nm Debye length in physiological buffers (length scale over which charge interactions are screened).

To study the extent of crowding posed by sialylation on live cell membranes, we examined human RBC membranes, where the average protein compositions and copy numbers are well characterized (21, 23). Approximately 23% of the RBC membrane surface area is occupied by proteins (24), with GYPA and Band 3 being two of the bulkiest and most abundant proteins. Given that GYPA contains 75% of the total sialic acids on RBCs, and because one-third to one-half of sugars on its 15 O-glycans are sialic acids (21), we hypothesized that GYPA plays a major role in mediating RBC surface crowding. Anionic transporter Band 3 is a multipass transmembrane protein with a single extracellular N-glycan with poly-LacNac (N-acetyllactosamine) glycans. Given a RBC surface area of 150 µm^2^, we estimate a surface density of 6,700/µm^2^ for Band 3 ( $5\times 10^{5}-10^{6}$ copies per cell) and 1,300/µm^2^ for GYPA ( $1\times 10^{5}-3\times 10^{5}$ per cell) (23, 25).

We found that the $K_{D}$ of the dextran 40k sensor is ~2.5× larger on the RBC surface compared to that on RBC surfaces treated with broad-spectrum serine protease, proteinase K (ProK) (Fig. 2*B*). We note that ProK treatment leads to only a partial digestion of the cell surface proteins, so the relative crowding state on the untreated wild-type cells are even larger when compared against bare lipid-coated beads (discussed below). To study the role of surface charges, we treated RBCs with sialidase and found that the $K_{D}$ decreased by 40% compared to the untreated RBC. This result may be surprising given that sialic acids occupy only ~1% of the total cell membrane by mass on RBCs (26). We observed negligible binding of fluorescently labeled *Sambucus nigra* lectin (binds preferentially to sialic acids in α-2,6 linkage) and *Maackia amurensis* lectin II (binds preferentially to sialic acids in α-2,3 linkage) on sialidase-treated RBC surfaces, verifying the proficient removal of sialic acids with sialidase. To verify that negative charge removal is the dominant mechanism of decreased $K_{D}$ , we used 1-Ethyl-3-(3-dimethylaminopropyl)carbodiimide (EDC) chemistry to neutralize the negative charges on the carboxylic group of sialic acids and other cell surface species (*Materials and Methods*). Consistent with our hypothesis, we found that even a partial neutralization of electrostatic charges reduced $K_{D}$ to a value comparable to the cells treated with sialidase (Fig. 2*B*). This further confirms that surface crowding cannot be described by surface density nor the protein molecular mass alone, and it demonstrates that surface charge can be a major contributor to crowding.

Because the Debye length is only 0.7 nm in physiological buffers (1), it may be surprising to observe such a strong effect of charge interactions. However, neighboring sialic acids on the O-glycans of GYPA are of the same order as the Debye length based on structural information (27), meaning that intra-chain charge interactions are still relevant. We note that the dextran sensors themselves contain a few charges due to the Alexa Fluor dyes conjugated to the dextran macromolecules, which can change the absolute magnitudes of $K_{D}$ and $K_{D}^{0}$ individually. However, only the relative difference between the crowded and bare surfaces matters, $K_{D}/K_{D}^{0}$ , so the crowding energy is unaffected as long as the normalized ratio is constant across different sensor chemistries, $\Delta U=k_{B}T\;ln\;\left(K_{D}/K_{D}^{0}\right)$ . As a control, we conjugated the dextran macromolecules to a charge-neutral 4,4-Difluoro-4-bora-3a,4a-diaza-s-indacene (BODIPY) dye, and found that the charges on the dye do not impact the binding kinetics nor crowding energies (*SI Appendix*). Electrostatic interactions between the sensors and the surface sialic acids are likely weak due to the larger separation distances and Debye screening between the dilute sensors and the sialic acids. We also performed control experiments in buffers with pH ranging from 6.0 to 8.0, and we did not observe changes to the binding of our dextran-based sensors, indicating that our results are not a pH-dependent effect from sialic acid removal. Our sensors were also unaffected by lipid-coated beads with 0 to 5 mol % of 1,2-dioleoyl-sn-glycero-3-phospho-L-serine, a lipid that carries a single negative charge on its head group. Lastly, to eliminate the possibility that sialic acid-binding proteins are bound to the cell surface and shield the membrane from sensor binding, we added exogenous sialic acid monosaccharides into the assay buffer to quench the sialic acid-binding proteins; we found no change in our results.

To understand the interplay between charge and glycocalyx architecture, we compared the average thickness of the RBC surface proteins on untreated and sialidase-treated RBCs. We used cell surface optical profilometry (CSOP) (16) to measure glycocalyx thickness by quantifying the height of a fluorophore conjugated to the N-terminus of surface proteins and fluorescent lectins attached to surface glycans (Fig. 2*C*). We found that the average heights of both proteins and glycans reduced by ~30 to 40% with sialidase treatment, consistent with the notion that the polyelectrolyte brushes deswell upon charge removal (22, 28). We tested the effects of charges in-silico by using MD simulations of surface-tethered polyelectrolytes interacting via a screened Coulomb (Yukawa) potential with two different electrostatic interaction distances, 1.0 and 2.0 nm. We found that a surface containing mucin-like glycoproteins swells by ~40% at surface polymer densities of ~15,000/µm^2^ (Fig. 2*D*), which is consistent with surface protein densities on cell membranes (21, 29). Our simulations support the hypothesis that the glycocalyx maintains a swollen architecture from electrostatic repulsion, and that the charged glycans pose a significant energy penalty against ligand binding. The mammalian cell surface contains approximately ~10^5^ to 10^6^ sialic acids/µm^2^, (1, 30, 31) which is further elevated in cancers (32, 33), supporting the idea that glycosylation can play an important role in modulating macromolecular binding to the cell surface.

### Cell Surface Crowding Is Significant and Varies across Different Cell Types.

**Fig. 3.**
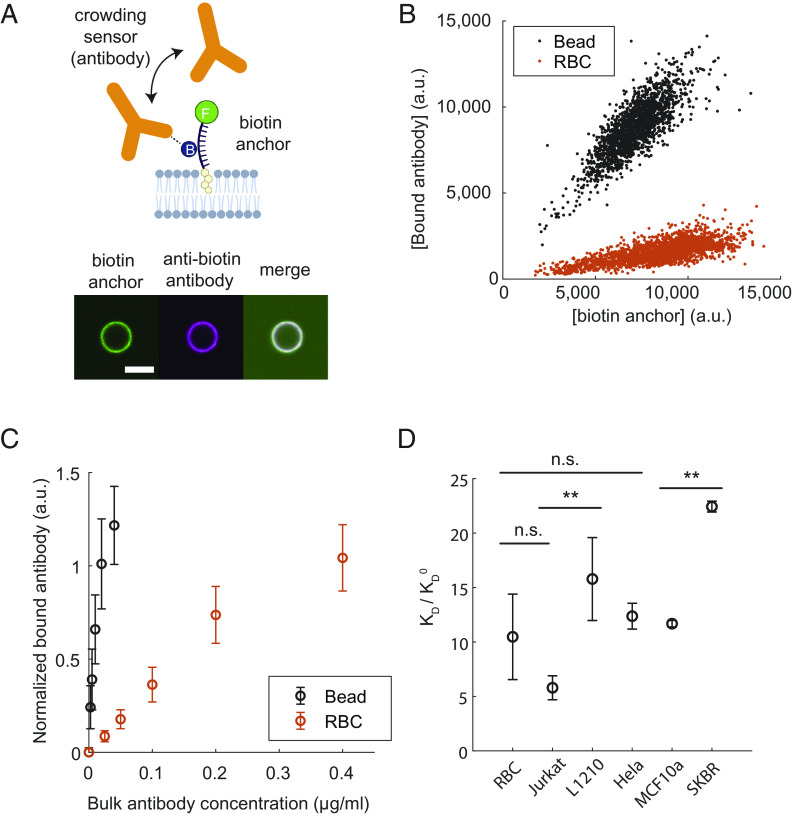
Cell surface crowding is significant and varies across different cell types. (*A*) Above: Schematic of the two-part crowding sensors that insert into the lipid bilayer with a biotin antigen presented above the membrane. The effective affinity of anti-biotin antibody to the biotin anchor is used as a reporter of surface crowding. Below: Florescence image of a lipid-coated bead with the FITC-biotin-cholesterol construct, anti-biotin antibody, and the merge. (Scale bar is 5 µm.) (*B*) Biotin anchor and the anti-biotin antibody bound on individual RBCs or beads for a fixed bulk antibody concentration, measured with flow cytometry. (*C*) Normalized surface concentration of bound antibody [data from panel (*B*) fitted with a linear slope] as a function of bulk antibody concentration. The ratio of the slopes at small antibody concentrations relative to that of the bare bead is used to determine the normalized dissociation constant on crowded surfaces, $K_{D}/K_{D}^{0}$ . Error bars represent the SD within the same sample. (*D*) Normalized dissociation constant of antibody, $K_{D}/K_{D}^{0}$ , on live suspension and adherent cells. Results are normalized by the binding affinity on bare lipid-coated beads, $K_{D}^{0}$ . To prevent internalization of the sensors into the cell interior, cells were incubated on ice throughout the measurement. Error bars represent the SD of the mean in three replicate measurements, except for HeLa and SKBR, which were measured twice. P-values are calculated based on an ANOVA test and Tukey's range test (n.s.: nonsignificant, * < 0.05, ** < 0.01, *** < 0.001).

Motivated by the unique insights obtained from our crowding sensors on human RBCs, we applied them to quantify cell surface crowding of other mammalian cells, including tumor cells with up-regulated glycosylation and sialylation (1). The affinity of our cholesterol-polymer sensors to the cell membrane is in part determined by the chemical affinity of the cholesterol tag with the lipid membrane. Therefore, differences in the native composition of the lipid membrane (particularly cholesterol content) may result in differences in the intrinsic affinity measurement that obscure the surface crowding contribution. While our dextran sensors with adjustable size are useful for comparing different crowding conditions within the same cell type, accurate comparisons between cell types are not possible due to different lipid membrane compositions. To overcome this challenge, we developed a crowding sensor that can measure across different cell types regardless of the cell size, shape, and lipid composition. The sensor consists of two parts—a cholesterol–biotin conjugate that incorporates into the cell membrane from solution (biotin anchor), and a fluorescent anti-biotin IgG antibody (crowding sensor) that measures cell surface crowding based on its binding affinity to the biotin anchor. The biotin anchor also contains a fluorescein 5-isothiocyanate (FITC) fluorophore via thymidine oligonucleotide linker to report its surface density on the membrane (5′-FITC-TTTTTT-biotin-TTT-cholesterol-3′) (Fig. 3*A* and *Materials and Methods*). The IgG size (~12 nm) (34, 35) is similar to our 40k dextran sensor (~10 nm). Control experiments on biotin-containing SLB beads showed a similar ~70% increase in $K_{D}$ of anti-biotin IgG on a 1% PEG3k brush compared to a bare surface, indicating that our IgG-based sensors are as sensitive to steric crowding as the dextran-40 sensors (*SI Appendix*). Our crowding sensor allows the simultaneous measurement of both the biotin surface density and anti-biotin antibody binding at single-cell resolution (Fig. 3*B*). At a given biotin anchor density, Fig. 3*B* shows that an IgG antibody binds much more readily on a bare bead surface compared to a RBC surface.

The FITC intensity on a cell surface is a readout of the saturating surface concentration of antibody binding, $C_{max}$ , which we use to plot the normalized bound antibody concentration (Fig. 3*C*). Any variations in cell size and surface area become normalized because the bound FITC and antibody intensity is linearly proportional to the cell size (*SI Appendix*). A readout of $C_{max}$ at single-cell resolution enables an accurate measurement of population heterogeneities within a cell sample, which cannot be accomplished using the dextran sensors. The ratio of the slope of the isotherm relative to the slope of the bare bead at small bulk antibody concentrations provides a measurement of $K_{D}/K_{D}^{0}$ . Technically, antibodies have two dissociation constants, $K_{D1}$ and $K_{D2}$ , corresponding to their bivalent binding. In general, the effective dissociation constant depends on a combination of two binding constants, and also the surface antigen concentration (36). Fortunately, at small antigen concentrations (less than ~1,400/µm^2^), the effective $K_{D}$ depends only on a single binding constant and is insensitive to the absolute antigen surface density (*SI Appendix*). At small sensor concentrations, we have verified that our results do not depend on the absolute concentration of the sensors on the surface.

We next compared dissociation constants for different suspension and adherent mammalian cells, normalized by the value on bare beads, $K_{D}^{0}$ (Fig. 3*D*). To prevent internalization of the macromolecules into the cell interior, cells were incubated on ice throughout the measurement. We confirmed using confocal microscopy that the sensors and antibodies are localized on the cell surface and bound homogeneously within the resolution limit (*SI Appendix*). We identified that all mammalian cells tested have surfaces that are significantly more crowded than any reconstituted bead surface evaluated in Fig. 1 (e.g., $K_{D}/K_{D}^{0}\approx 22$ for SKBR breast epithelial tumor cells compared to $K_{D}/K_{D}^{0}\leq 2$ on bead surfaces with PEG3k, Fig. 1*E*). This highlights the importance of using native cell membranes to study the physiological impacts of cell surface crowding. As shown in Fig. 3*D*, we observed large variations in crowding across different cell types. For example, SKBR cells displayed 2× more crowding compared to MCF10a cells, which are nonmalignant breast epithelial cells commonly used to model normal breast epithelia behavior. Many mammalian cell surfaces contain a large concentration of sialic acids, which can locally reduce the pH due to its negative charge (1). Because the IgG antibodies are charged macromolecules (unlike our dextran sensors), there may be a pH-dependent binding affinity of IgG onto charged surfaces (37–39). If desired, one could use our neutral dextran sensors within the same cell type to isolate the crowding effects specifically due to charge, as we demonstrated with RBCs.

### Cell Surface Crowding Is Altered by Surface Protein Overexpression and Oncogenic Mutations.

**Fig. 4.**
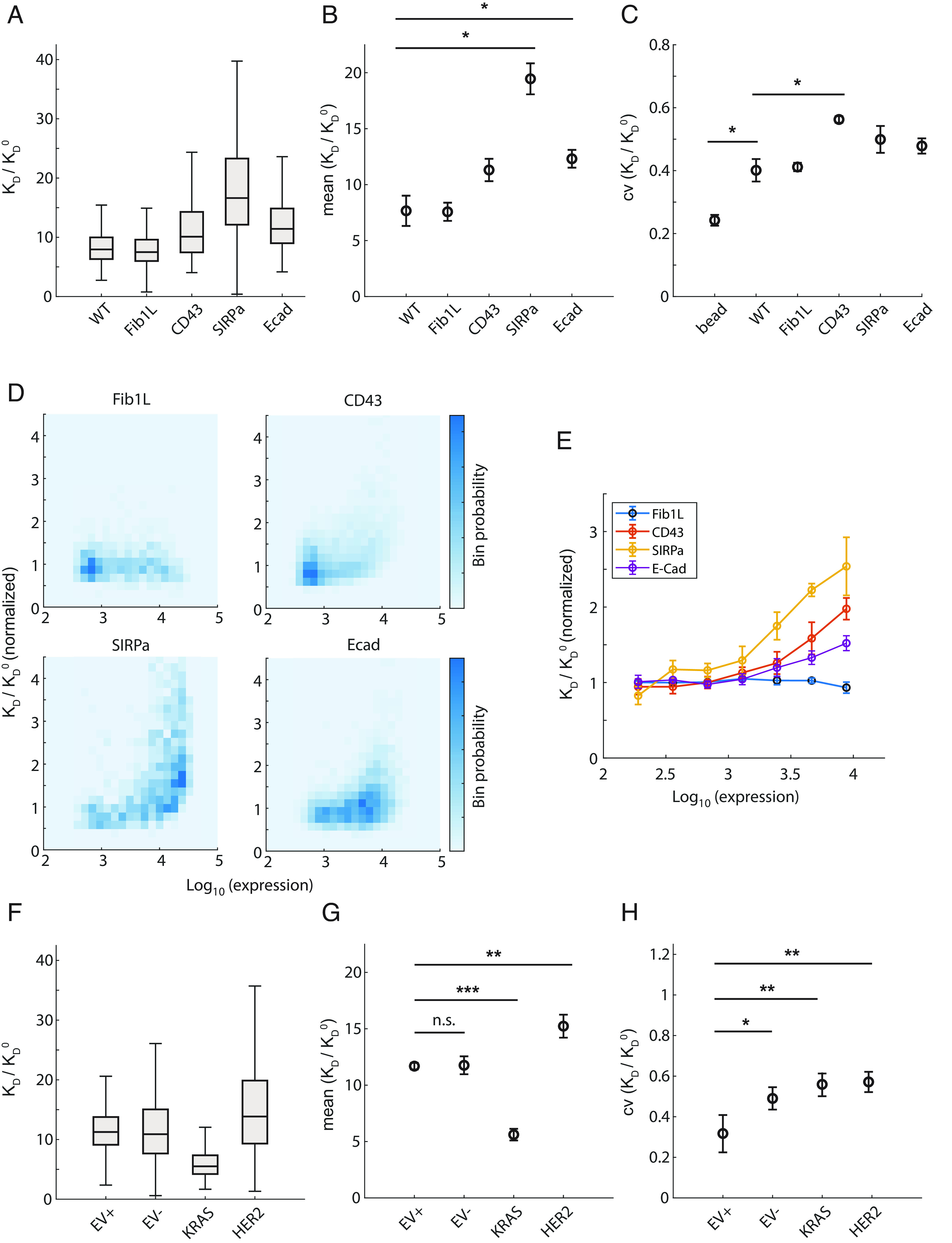
Mutations and surface protein overexpression alter cell surface crowding and variability. (*A*) Reduction in antibody binding on cells overexpressing various proteins. The distribution is represented as a box and whisker plot showing the median, lower and upper quartiles, and minimum and the maximum values ignoring outliers. To prevent internalization of the sensors into the cell interior, cells were incubated on ice throughout the measurement. N > 300 cells are measured in all conditions in a single run. (*B* and *C*) The mean and coefficient of variance (CV) of affinity change obtained from two replicate measurements. *P*-values are calculated based on an ANOVA test and Tukey's range test (* < 0.05). (*D*) Reduction in antibody-binding affinity as a function of protein overexpression in a bin scatter plot. (*E*) Reduction in antibody-binding affinity as a function of protein overexpression. Error bars represent the SD of the mean from three replicate runs. (*F*) Reduction in antibody-binding affinity in MCF10a and MCF10a transformed with KRAS(G12V) and HER2. The distribution is represented as a box and whisker plot showing the median, lower and upper quartiles, and minimum and the maximum values ignoring outliers. N > 300 cells are measured in all conditions in a single run. EV+: empty vector control supplemented with growth factors. EV-: empty vector control without growth factors. (*G* and *H*) The mean and CV of affinity change obtained from three replicate measurements. *P*-values are calculated based on an ANOVA test and Tukey's range test (* < 0.05, ** < 0.01, *** < 0.001).

After observing significant differences in cell surface crowding across different cell types, we asked whether changes in protein expression can alter crowding within a single cell type. To test if our crowding sensor could resolve such changes, we used lentivirus to generate HEK cell lines expressing or overexpressing surface proteins of different heights. Our measurements show that the surface crowding of cells expressing CD43, signal regulatory protein alpha (SIRPɑ), and E-cadherin increased crowding, while the crowding of Fib1L-expressing cell remains approximately the same (Fig. 4*A*). Interestingly, SIRPɑ expression increased crowding by approximately 2.5× compared to the wild-type (Fig. 4*B*) and cell–cell crowding variability by ~25% (Fig. 4*C*), whereas CD43 expression increased crowding by less than a factor of 2× (Fig. 4*B*) and cell–cell crowding variability by ~40% compared to the wild type (Fig. 4*C*). We note that the crowding variability measured in the wild type is ~60% larger than that in beads (Fig. 4*C*). This suggests that expression changes within a cell population can lead to both increased mean and variance in crowding. A key advantage of our antibody probe is its ability to measure crowding at single-cell resolution and to obtain a distribution of crowding within a cell population. In the results presented prior to this section, we have focused on the mean surface crowding over an entire cell population. Our results in Fig. 4*C* demonstrate that the variance of the crowding distribution also contains valuable information about the heterogeneities within a cell population.

To further examine the extent of surface crowding observed in different cell lines, we expressed the same surface proteins with a small, 12 residue Spot-tag fused at their N-terminus (16). We measured the crowding sensor binding as a function of protein expression level by quantifying the protein surface density using a fluorescent anti-Spot nanobody (V_HH_), a small ~2 to 4-nm antibody fragment. We found that crowding increased dramatically as a function of protein overexpression level of CD43, SIRPɑ, and E-cadherin (Fig. 4*D*). We binned the cells by protein expression level and calculated crowding for each bin, which revealed that SIRPɑ increases the crowding the most, followed by CD43 and E-cadherin, while for Fib1L, little change was observed over 2 orders of magnitude of expression (Fig. 4*E*). The extracellular domains of Fib 1L, SIRPɑ, CD43, and E-cadherin, are 89, 317, 204, and 543 amino acids, respectively, underscoring that surface crowding is determined not only by protein molecular mass but by physical properties such as surface charge and structure. Taken together, these results demonstrate that changes in a single protein expression level can significantly alter the total cell surface crowding.

We next tested whether oncogenic transformation alters cell surface crowding using the breast epithelial cell line MCF10a, expressing the common oncogenes HER2 and KRAS(G12V) (40). MCF10a requires growth factors for proliferation but can also maintain homeostasis in the absence of growth factors. We found that MCF10a cells exhibit similar levels of crowding in both proliferating and nonproliferating states (Fig. 4 *F* and *G*). We measured the effect of the oncogenes and found that KRAS-expressing cells decreased crowding by 2× while HER2-expressing cells show slightly increased crowding (Fig. 4*G*). Interestingly, the variance of cell surface crowding was increased in transformed cells when compared to that of EV+, the state of non-malignant growth (Fig. 4 *F* and *H*). Consistent with this, KRAS(G12V) and HER2 expression in MCF10a cells is known to significantly alter the surfaceome as well as glycosylation pattern (41). Furthermore, surface protease activity also changes in KRAS and HER2 cells, cleaving different surface protein groups (42). Our observations suggest that the molecular-level changes in transformed cells result in collective biophysical changes in their cell surface.

## Discussion

Traditional biochemical, genetic, and proteomics approaches excel at characterizing the molecular features of membrane proteins (29, 43–46). However, these approaches cannot capture the multibody biophysical interactions on cell surfaces that give rise to crowding. As a result, mechanistic understanding of the biophysical interactions that modulate the organization of the cell surface glycocalyx has been limited. In this work, we developed a simple experimental technique to quantify the impact of cell surface protein glycosylation, density, charge, stiffness, and other physical properties on macromolecular binding to the plasma membrane of live cells.

Our measurements provide a method to quantify the steric energy penalty posed by a crowded cell surface. We found that these energies correspond to ~0.75 to 3 k_B_T for the case of IgG binding to buried receptors. These results provide a perspective on what cell surface “crowding” means and how it might be quantified. The free energy posed by the crowded cell surface arises from an osmotic pressure generated by the glycocalyx, given by $\Delta U\approx \Pi V^{eff}$ , where Π is the osmotic pressure and $V^{eff}$ is the effective volume occupied by the macromolecule within the glycocalyx (*SI Appendix*). The free energy may be interpreted as the mechanical work to displace a volume $V^{eff}$ inside a crowded environment with pressure Π . All crowding contributions are captured by the osmotic pressure, including protein glycosylation, density, charge, stiffness, and other physical properties. In *SI Appendix*, we demonstrate using MD simulations that all sensor-binding data collapse onto a universal curve described by a “cell surface equation of state”. We propose that the osmotic pressure is a universal metric that acts as a quantitative reporter of cell surface crowding, as opposed to other proxy metrics like protein molecular weight, surface charge, or number density.

Based on our measurements, the cell surface osmotic pressures are given by $\begin{array}{c} \Pi =\Delta U/V^{eff}=k_{B}T\;\ln\;\left(K_{D}/K_{D}^{0}\right)/V^{eff}=1-4\;\mathrm{kPa}, \end{array}$  based on $K_{D}/K_{D}^{0}\approx 2-20$ and an IgG that excludes a volume based on its size of 10 nm. Note that these absolute pressure values are estimates because an accurate value of the sensors’ excluded volume in the glycocalyx is unknown. These approximate values are consistent with the pressures generated by steric crowding interactions between synthetic polymer brushes on membranes, which are sufficient to bend the lipid bilayer (47–49). Interestingly, the surface osmotic pressures measured by our crowding sensors are larger than the stiffness of the cell cytoplasm (~100 Pa), the cell cortex (~1 kPa), and the thick glycocalyx of endothelial cells (100 to 500 Pa), as measured using atomic force microscopy with a large bead tip (50, 51). We anticipate that the 1 to 4 kPa pressures are highly localized just above the membrane surface and that these pressures decay rapidly as a function of distance from the surface into the bulk fluid. Yet, large soluble ligands, antibodies, viruses, and receptors from opposing cell surfaces that bind close to the membrane surface will experience large repulsive pressures. Indeed, we found that the surface crowding can reduce the binding affinity of an IgG antibody to a cell surface by 20×.

We end by noting several areas for future investigation. First, we focused on crowding measurements at a fixed distance very close to the membrane surface. Since the glycocalyx is a fully three-dimensional structure, we anticipate that the surface crowding is height dependent and a measurement of crowding as a function of distance from the membrane may provide further insight into spatial organization. Second, our sensors may be used to study temporal changes in surface protein expression during different stages of the cell cycle, tumor progression, cell senescence, and cell differentiation. Third, while we focused on the extracellular side of the cell membrane, the cytoskeleton on the interior side plays a key role in membrane protein organization, and the interplay between cortical actin organization and glycocalyx crowding mediated by actin-binding proteins merits further study. Fourth, we envision the use of our technique on more complex systems beyond immortalized, individual cells grown on artificial surfaces. If appropriate contrasting agents and reporter tags can be functionalized on our sensors, alternative imaging modalities like ultrasound, X-ray, and MRI may allow a quantification of cell surface crowding in complex systems, like deep tissues and organoids. Lastly, despite the advances in quantitative transcriptomics and proteomics (29, 43–46), it remains unclear whether and how protein copy number on a cell surface is regulated under physiological conditions or whether surface crowding is simply an unregulated outcome of protein expression, glycosylation, and trafficking. Our crowding sensors may be used to address these and other topics related to the biophysical properties and collective function of the cell surfaceome.

## Materials and Methods

### Materials.

See *SI Appendix* for a listing of materials and resources used for this work.

### Protein Purification.

Multi-FN3-domain proteins containing C-terminal 10× His-tag and N-terminal ybbR-tags were purified as described previously (20). Briefly, FN3 proteins were expressed in Rosetta DE3 cells (EMD Millipore), lysed by sonication, and purified over a His-Trap HP column (GE Healthcare). The proteins were gel-filtered and their size was confirmed via a Superdex 200 column on an AKTA Pure system (GE Healthcare).

### Microscopy.

All imaging was carried out on an inverted Nikon Eclipse Ti microscope (Nikon Instruments) equipped with a Yokogawa CSU-X spinning disk using an oil-immersion objective [Apo TIRF 60× and 100×, numerical aperture 1.49, oil]. Three solid state lasers were used for excitation: 488 nm, 561 nm, and 640 nm (ILE-400 multimode fiber with BCU, Andor Technologies). The laser power at the sample plane was less than 1.5 mW for all three channels. Fluorescent light was spectrally filtered with emission filters (535/40 m, 610/75, and 665LP, Chroma Technology) and imaged on a sCMOS camera (Zyla 4.2, Andor Technologies). For CSOP measurements, Z-stack was acquired using a piezo z-stage (nPoint, Inc.).

### Synthesis of Crowding Sensors.

Cholesterol N-hydroxysuccinimide (NHS) (Nanocs) was dissolved in a 1:2 ratio of ethanol and dimethylsulfoxide (DMSO). Amino 10k dextran and 40k dextran (Invitrogen) and Cholesterol-PEG-amine (Creative PEGWorks, Inc; 966 g/mol MW) were dissolved in DMSO. Equimolar ratio of NHS-dye (choice of AF488, AF555, AF647, or BODIPY), and cholesterol NHS were mixed at a 10:1 molar ratio with cholesterol-PEG-amine, 5:1 molar ratio with 10k dextran amino, 20:1 molar ratio with 40k dextran amino, and left overnight at 50 °C. Control probes without cholesterol conjugation were mixed without cholesterol NHS. To remove unreacted NHS reactants, the reaction mixture was processed through a Zeba Spin Desalting Column, 7K MWCO (Thermo Scientific). The labeling ratio of cholesterol and dye was recorded using a NanoDrop 2000c spectrophotometer (Thermo Scientific).

Small aliquots were stored in −80 °C and used within a few months. Thawed sensors were used within the same day. The labeling ratio of the small sensors is 1 cholesterol/dye, 10k sensor is ~3 cholesterol/dye, and 40k sensor is ~7 cholesterol/dye. The approximate hydrodynamic diameters of the Chol-PEG-dye, dextran 10k, and dextran 40k sensors are ~2, 4, and 10 nm, respectively (19). As a control, we synthesized dextran sensors without the cholesterol anchors and observed no binding to membrane surfaces, verifying that nonspecific interactions between the dextran–dye conjugate and the membrane is negligible (*SI Appendix*).

### Preparation of SLB on Glass Beads.

Lipid-coated glass beads were created by coating glass microbeads with a fluid SLB. Small unilamellar vesicles (SUVs) were prepared by rehydrating a lipid sheet composed of 1,2-dioleoyl-sn-glycero-3-phosphocholine (DOPC) and other phospholipids with pure deionized water. For SLBs involving the attachment of His-tagged purified proteins, DOPC lipids were mixed with 7.5% of DGS-Ni-NTA. For SLBs involving the binding of anti-biotin antibody, DOPC lipids were mixed with 1% biotinyl cap phosphoethanolamine (PE) and 1% of PEG3k PE. After rehydrating for 30 min, the solution was vigorously vortexed, sonicated at low power (20% power) using a tip-sonicator (Branson Sonifier), and finally filtered through a 0.2-mm filter (Millipore). Stock solutions of SUVs were stored at 4C and were used within 48 h to avoid phospholipid oxidization.

Then, 4.07-μm and 6.46-μm glass micro-bead (Bangs labs) slurry (10% solids) were cleaned using a 3:2 mixture of H_2_SO_4_:H_2_O_2_ (Piranha) for 30 min in a bath sonicator, and were spun down at 1,000 g and washed 3 times before being resuspended in pure water. Clean beads were stored in water at room temperature and used within 48 h. To form SLBs, 45 μL SUV solution was added with 5 μL 10× 3-(N-morpholino)propanesulfonic acid (MOPS) buffer (500 mM MOPS pH 7.4, 1 M NaCl) and 10 μL clean bead suspension, and mixed gently. The bead/SUV mixture was incubated for 15 min at room temperature while allowing the beads to sediment to the bottom of the tube. Beads were washed 5 times with 4-(2-Hydroxyethyl)-1-piperazine ethanesulfonic acid (HEPES) buffer (50 mM HEPES pH 7.4, 100mM NaCl) by gently adding/removing the buffer without resuspending the glass beads into solution.

For experiments involving poly His-tagged purified Fibcon and GYPA proteins, 200 nM protein was added into the DGS-Ni-NTA lipid-coated bead solution and incubated at room temperature for 20 min. Beads were washed 3 times with HEPES buffer by gently adding/removing the buffer without resuspending the glass beads into solution. For sialidase-treated GYPA beads, the beads were further treated with 200 mUn/mL sialidase for 30 min at 37 °C. For ProK treated beads, the beads were treated with 0.05 mg/mL ProK for 20 min at 37 °C. Beads were washed 3 times with HEPES buffer. GYPA proteins were fluorescently labeled with NHS-Alexa Fluor 555, and we used confocal microscopy and flow cytometry to verify that the GYPA surface density did not change before and after sialidase treatment.

### Preparation of RBCs.

Becton Dickinson Microtainer contact-activated lancet was used to withdraw blood from volunteers. A small amount (10 μL) of blood was washed in phosphate-buffered saline (PBS) with 2 mM ethylenediaminetetraacetic acid to remove soluble proteins and plasma from whole blood. RBCs were stored in PBS solution at 4 °C and used within a day. All procedures followed a UC Berkeley IRB approved protocol (CPHS Protocol Number: 2019-08-12454).

To cleave sialic acid from the surface of RBCs, sialidase was used at 50 mUn/mL in PBS at 37 °C for 2 h. No protease activity was found in our stock sialidase (*SI Appendix*). To digest the RBC surface proteins, ProK was added at 0.05 mg/mL in PBS at 37 °C for 1 h. To remove negative charges on carboxylic acid groups on RBC surfaces, 1 mM hydrazide-biotin and 30 mM EDC were mixed with untreated cells in PBS at room temperature for 3 h. The cell surface was visualized with confocal microscopy using fluorescently labeled streptavidin (*SI Appendix*).

RBC measurements were conducted using the synthetic dextran sensors. The small Chol-13xPEG-488 sensor was varied between 0 and 10 nM, the medium 10k-555 sensor was varied between 0 and 15 nM, and the large 40k-647 sensor was varied between 0 and 5 nM.

### Flow Cytometry.

An Attune NxT Acoustic Focusing Cytometer (ThermoFisher Scientific) was used for all flow cytometry experiments. For RBC measurements, we added $\approx \;10^{6}$ RBC into 1 mL of PBS containing appropriate sensors (typically ≈30 μL stock RBC, containing 10 μL of collected blood in 400 μL of PBS).

Since the crowding energy is given by $\Delta U=k_{B}T\;\ln\;\left(K_{D}/K_{D}^{0}\right)$ , the important quantity is the ratio of the dissociation constants on a crowded and bare surface, $K_{D}/K_{D}^{0}$ . The absolute magnitudes of $K_{D}$ for our sensors are unimportant and irrelevant for measuring crowding. We therefore obtain the slope of the bound sensor on a crowded surface at small bulk sensor concentrations, and normalize this value by the slope on bare lipid-coated beads. This provides a direct measurement of the relative difference, $K_{D}/K_{D}^{0}$ , if the bound saturation concentration, $C_{max}$ , is equal on the crowded and bare surfaces: $K_{D}/K_{D}^{0}=\left(\theta /C_{bulk}\right)/\left(\theta^{0}/C_{bulk}\right)\approx \left(C/C_{bulk}\right)/\left(C^{0}/C_{bulk}\right)$ if $C_{max}\approx C_{max}^{0}$ . To verify this approach, we performed a control experiment across larger sensor concentration to obtain the full isotherm curve and found similar results for the ratio $K_{D}/K_{D}^{0}$ . In the control experiments, the maximum saturating concentrations of the sensors, $C_{max}$ , were obtained by incubating the beads and RBCs with 0.5 µM bulk sensor concentration. The bound sensor concentrations were normalized by the saturation value to obtain the fractional surface coverage, $\theta =C/C_{max}$ . The slope at small bulk concentrations was used to obtain the dissociation constant. A representative dataset containing the full isotherm for the dextran 40k sensor is shown in *SI Appendix*.

For our antibody-based crowding measurements, we first incorporated a FITC-biotin-DNA-cholesterol construct (5′-FITC-TTTTTT-biotin-TTT-cholesterol-3′) into the cell membrane. Therefore, the saturating surface concentration, $C_{max}$ , is set by the total FITC-biotin-DNA-cholesterol inserted into the cell membrane, which we measure directly from the FITC intensity. Because we used small concentrations of the construct in the cell membrane, there was never an excess of unbound antigen at saturating concentrations. As evidence of this statement, we found during our experimental design that the FITC signal reduces to zero when adding a saturating concentration of anti-FITC antibodies in the bulk, which shows that all FITC sites were occupied and quenched due to binding.

Since the synthetic crowding sensors and the monoclonal antibodies are fluorescently labeled, the intensity units from the flow cytometer report the bound sensor concentration. The fractional surface coverage is defined as $\theta =C/C_{max}$ , where C is the surface concentration of the sensor on a given sample averaged across ~20,000 events. This fractional surface coverage is plotted as function of bulk concentration of the sensor, and the slope at small bulk sensor concentrations gives the dissociation constant. Since only the ratio $K_{D}/K_{D}^{0}$ is needed for the crowding energy (as opposed to their individual values), we compare the slopes of the normalized antibody signal from the flow cytometer (antibody channel intensity divided by the FITC channel intensity). We do not need to convert the intensity units into real concentration units because the conversion factor would normalize out when taking the ratio of the slopes. If absolute magnitudes of the individual dissociation constants are needed, fluorescence intensities can be converted to surface concentrations using Quantum Molecules of Soluble Fluorochrome kits (Bangs labs).

We allowed the measurement probes (dextran and biotin antibody) to reach equilibrium on the membrane surfaces for 45 min prior to flow cytometry analysis. A representative time trajectory of sensor binding on RBCs is shown in *SI Appendix*.

In this work, all replicates involving cells indicate biological replicates, in which cells were independently grown and measured on different days.

### CSOP Height Measurements on RBCs.

PBS solution containing unconjugated wheat germ agglutinin (WGA) was incubated into an 8-well chambered cover glass (Cellvis; catalog number: C8-1.5H-N) to coat the glass with WGA. This helps to immobilize the RBCs when added to the chamber. The chambers were washed and filled with 0.25× PBS in Milli-Q water to swell the RBC into a bloated sphere. Our Chol-13×PEG-488 sensors were added (83 nM) as a membrane reporter. Untreated and sialidase-treated RBCs were added to separate wells. We acquired approximately 15 slices of images straddling the bead equator with a 100-nm step size. Each z-stack image contained up to ten beads per field-of-view. It is critical to locate the equator in both channels to ensure that accurate offsets are calculated (16). More than 100 RBCs were acquired and processed in our custom MATLAB script. Cells with visible defects, including nonspherical shape, membrane tubule and/or bud formation were removed from analysis. In addition, chromatic aberration and other optical offsets were subtracted from our signal by taking a baseline calibration measurement on RBC using our membrane sensor pairs Chol-13xPEG-488 at 83 nM and Chol-13×PEG-647 at 125 nM. The height of the cell surface proteins is obtained by quantifying the difference between the fluorescently labeled height and the aberration offset, $\langle h\rangle \;=\;\langle h_{\mathrm{measured}}\rangle -\langle h_{offset}\rangle$.

The N-terminal alpha-amines of RBCs were labeled with DyLight 650-4×PEG NHS at 100 μM for 15 min at room temperature in PBS at pH 6.5 (with citric acid). NHS reaction at low pH facilitates preferential labeling of the N terminus (average $pK_{a}\approx 5-7$ ) as opposed to other aliphatic amines on the protein (average $pK_{a}\approx 10.5$ ) and phosphoethanolamine lipids ( $pK_{a}>10$ ) (52). The reaction mixture was washed with PBS to remove excess NHS reagent. Proteinase-K treatment of these cells showed that the majority of the label was removed from the RBC surface, verifying that the NHS reaction predominantly labeled the proteins and not the lipids. For sialidase treatment, the cells were treated with 50 mUn/mL for 1 h at 37 °C to digest sialic acids from the cell surfaces prior to CSOP measurement. No divalent cations (including calcium and magnesium) were added in the measurements, which could result in unwanted cross-linking between negative charges in the glycocalyx.

In addition to N-terminal labeling of RBC surface, we also used lectins as a readout of cell surface height. For height measurements based on lectins, fluorescein labeled *Agaricus bisporus* lectin (ABL), and *Erythrina cristagalli* lectin (ECL) were used. ABL binds to galactosyl (beta-1,3) N-acetylgalactosamine (also called the Thomsen-Friedenreich antigen, or T disaccharide), which are heavily expressed on GYPA proteins on the RBC surface. Unlike peanut agglutinin, which does not bind sialylated T antigen, ABL binds either sialylated or asialylated forms, which makes them good markers for height measurements. Although we noticed a 30 to 50% increase in the binding of ABL to sialidase-treated RBCs, we still observed a strong signal on the fully sialylated surface and assumed that the spatial distribution of bound lectins does not change significantly between untreated and sialidase-treated RBCs. ECL binds to the disaccharide Gal (*β*1-4) GlcNAc, called LacNAc. Although ECL does not bind to LacNAc terminated with sialic acids, we still observed strong binding on RBCs for all conditions. The single N-glycosylation on the Band3 protein contains LacNAc, which is abundant on the RBC surface. The single complex N-glycan on Band3 is heterogeneous in size, based on the variable repeats of poly-LacNAc units (Gal-*β*1→4 GlcNAc *β* 1→3). The end of the oligosaccharide can be linked to sialic acid, fucose, or left exposed.

### Coarse-Grained MD Simulations of Protein Polymers and Sensors.

To construct a model of macromolecular transport across cell surface proteins and glycocalyx, we performed coarse-grained MD simulations of particle transport within semi-flexible polymers diffusing on two-dimensional surfaces. See *SI Appendix* for a detailed explanation of our simulations. Briefly, we model surface proteins using a Kremer–Grest bead-spring model (53), with each bead representing a structured protein domain or a coarse-grained unit of an intrinsically disordered domain. In this work, the membrane does not deform nor fluctuate in the out-of-plane dimension, although these effects may be included. Simulations were performed using a graphics processing unit-enabled HOOMD-blue MD package (54). A dilute concentration of soluble particles with different sizes were added to model the dynamics of sensor binding to the cell surface. The diameters of the Chol-PEG, 10k, and 40k sensors were modeled with spheres of diameter 3, 5, and 10 nm, respectively.

### Mammalian Cell Culture and Preparation.

- HEK293T cells were obtained from University of California, San Francisco (UCSF) Cell Culture Facility and grown in Dulbecco’s Modified Eagle Medium (DMEM) (Life Technologies) supplemented with 10% heat-inactivated Fetal Bovine Serum (FBS) (Life Technologies) and 1% Pen-Strep (Life Technologies), at 37 °C, 5% CO2.
- SKBR3 cells were purchased from American Type Culture Collection (ATCC) and grown in McCoy’s 5A (ATCC) supplemented with 10% FBS and 1% Pen-Strep.
- Jurkat and L1210 cells were purchased from American Type Culture Collection (ATCC) and is grown in Roswell Park Memorial Institute (RPMI) (Life Technologies) supplemented with 10% FBS, 1% Pen-Strep.
- HeLa cells were obtained from University of California, Berkeley Cell Culture Facility and grown in DMEM (Life Technologies) supplemented with 10% heat-inactivated FBS (Life Technologies) and 1% Pen-Strep (Life Technologies), at 37 °C, 5% CO2.
- MCF10a-derived cell lines expressing oncogenes were a kind gift from Kevin Leung and James Wells at UCSF (41).

For depletion experiments, cells were grown in DMEM supplemented with 5% horse serum, 0.5 mg/mL hydrocortisone, 100 ng/mL cholera toxin, and 1% Pen-Strep. For proliferation experiments, media were further supplemented with 20 ng/mL epidermal growth factor and 10 μg/mL insulin. Cells were passaged by treatment with 0.05% trypsin, and for depletion experiments, by treatment with versene. Cells were passaged every 2 to 3 d. One day prior to flow cytometry experiments, care was taken to seed adherent cells at dilute to intermediate concentrations to prevent the formation of multicell clusters. For suspension cells (RBCs, L1210, Jurkat), cells were washed by centrifuging at 150 g for 1 min and resuspending in PBS to remove media. Adherent cells were carefully scraped from the plastic substrate using a cell scraper and then washed.

To prevent internalization of the sensors into the cell interior, cells were then incubated on ice for 10 to 15 min, and all subsequent steps were performed on ice. 10 to 100 nM of the FITC-biotin-cholesterol sensor was incubated with the cells for 20 min. The final isotherm for the anti-biotin antibody is insensitive to the precise amounts of biotin anchor on the cell surface, as long as the total concentration is low (less than ~1,400/µm^2^), as evidenced by the fixed slope of the biotin anchor vs. antibody intensity in the raw flow cytometry data (Fig. 3*B*). Cells were washed thoroughly (5×) in PBS in a centrifuge set to 4C. Finally, the cells were added into tubes prepared with several different Alexa Fluor 647-labeled anti-biotin monoclonal antibody concentration (0 to 0.5 μg/mL). After incubation on ice for 45 min, the samples were measured on the flow cytometer.

### Lentiviral Preparation and Cell Line Generation.

Lentivirus was produced by transfecting the transfer plasmids, pCMV-dR8.91, and pMD2.G (1.5 µg, 1.33 µg, and 0.167 µg per 35 mm well) into 293T cells grown to approximately 80% confluency using Mirus TransIT-293 Transfection Reagent (Promega) per manufacturer protocol. After 60 to 72 h, supernatant containing viral particles was harvested and filtered with a 0.45-μm filter. Supernatant was immediately used for transduction or aliquoted and stored at −80 °C. Cells were seeded at 20% confluency in 35-mm dishes and 0.5 to 1 mL filtered viral supernatant was added to the cells. Medium containing virus was replaced with fresh growth medium 24 h postinfection. Infected cells were imaged to assess transduction efficiency and then used in flow cytometry assays as described above.

## Supplementary Material

Appendix 01 (PDF)

## Data Availability

All study data are included in the article and/or *SI Appendix*. Raw data files for the main results are available at a publicly accessible database (https://ucsb.box.com/s/1l8bhfbdbtw156kwp2w0drbp1m48oc0s) (55). These data will be accessible upon publication.
