# Supplementary material for: Engineered molecular sensors for quantifying cell surface crowding
Source: Proc Natl Acad Sci U S A. 2023 May 15;120(21):e2219778120. doi: 10.1073/pnas.2219778120 (PMC10214205; doi:10.1073/pnas.2219778120)
Supplement: Supplementary file 1 — Appendix 01 (PDF) [file pnas.2219778120.sapp.pdf]

Supporting Information

Engineered molecular sensors for quantifying cell surface crowding

Authors: Sho C. Takatori[a,e,1,2], Sungmin Son[a,f,1,2], Daniel S.W. Lee[a], and Daniel A. Fletcher[a,b,c,d,2]

[a] Department of Bioengineering, University of California, Berkeley, Berkeley, CA 94720, USA
[b] UC Berkeley/UC San Francisco Graduate Group in Bioengineering, Berkeley, CA 94720, USA
[c] Division of Biological Systems and Engineering, Lawrence Berkeley National Laboratory, Berkeley, CA 94720, USA;
[d] Chan Zuckerberg Biohub, San Francisco, CA 94158, USA
[e] Department of Chemical Engineering, University of California, Santa Barbara, Santa Barbara, CA 93106, USA.
[f] Department of Bio and Brain Engineering, KAIST, Daejeon, Republic of Korea.

[1] S.C.T. and S.S. contributed equally to this work.

[2] To whom correspondence should be addressed. E-mail: stakatori@ucsb.edu ; smson@kaist.ac.kr ; fletch@berkeley.edu

Chapter I. Dynamics of macromolecular binding on crowded surfaces

The goal of this chapter is to develop a theory of macromolecular adsorption to identify a quantitative metric of surface crowding. We aim to obtain a direct relationship between surface binding of soluble macromolecules and the crowding state of the surface, including the effects of variable glycoprotein length, polydispersity, stiffness, density, charge, and all other interactions. We propose that the osmotic pressure is a universal metric that acts as a quantitative reporter of cell surface crowding, as opposed to other proxy metrics like protein molecular weight or number density.

1. Momentum balance on crowded cell membrane surfaces

The Cauchy momentum balance in a control volume above the cell surface is given by

$$\rho \frac{D\boldsymbol{u}}{Dt} + \nabla \cdot \boldsymbol{\sigma} + \boldsymbol{b} = \boldsymbol{0}, \tag{1.1}$$

where D/Dt is a material derivative, $\boldsymbol{u}$ is the suspension average velocity, $\boldsymbol{\sigma}$ is the total stress of the suspension, and $\boldsymbol{b}$ is an external force. Incompressibility further requires that $\nabla \cdot \boldsymbol{u} = 0$. The total suspension stress, which include both the fluid and the surface polymers, is given by

$$\boldsymbol{\sigma} = -p_f \boldsymbol{I} + 2\eta_0(1 + 5\phi/2)\boldsymbol{E} + \boldsymbol{\sigma}^{(P)}, \tag{1.2}$$

where p_f is the fluid pressure, η_0 is the viscosity of the continuous Newtonian solvent, $\boldsymbol{E}$ is the rate-of-strain tensor, ϕ is the volume fraction of particles (= $4\pi a^3 n/3$ for spheres), n is the number density, $5\phi/2$ is the Einstein shear viscosity correction that is present for all suspensions, and the particle contribution to the stress $\boldsymbol{\sigma}^{(P)} = -nk_\mathrm{B}T\,\boldsymbol{I} + \boldsymbol{\sigma}^P$, where $\boldsymbol{\sigma}^P = -n\langle \boldsymbol{x}_{ij}\boldsymbol{F}_{ij}\rangle$ is the virial stress contribution from interparticle interactions. The force $\boldsymbol{F}_{ij}$ includes all interparticle interactions between the particles.

At low Reynolds numbers and in the absence of external forces, the momentum balance of the suspension (solvent plus any particles and polymers) at equilibrium is

$$\nabla \cdot \boldsymbol{\sigma} = \boldsymbol{0}. \tag{1.3}$$

It is important to note that an aqueous polymer brush surface is at minimum a 2-component system: the fluid plus the particles (polymers). Therefore, the total suspension stress includes contributions from both the solvent and the particles (polymers), $\boldsymbol{\sigma} = -p_f\boldsymbol{I} + \boldsymbol{\sigma}^{(P)}$. Therefore, at every continuum point in the suspension, the overall pressure is a sum of the local fluid pressure, p_f, and the osmotic pressure from interactions among the particles within the suspension, including polymers, free ions, and other interacting particles, $\Pi = -\mathrm{tr}\,\boldsymbol{\sigma}^{(P)}/3$ (see Fig. S1.1). If there are free ions in solution, then those electrolytes would generate an additional osmotic pressure that is included within Π via the stresslet $\langle \boldsymbol{x}_{ij}\boldsymbol{F}_{ij}\rangle$. Assuming that there are no fluid velocities and that the system is translationally invariant (i.e., independent of in-plane coordinates x and y), Eq. (1.3) becomes $\partial\sigma_{zz}/\partial z = 0$. This reveals that the total suspension stress is constant everywhere along the z-direction (normal to the membrane surface).

Using Fig. S1.1, we shall conduct a momentum balance to clarify the relationship between the osmotic pressure and swelling of the polymer brush. Suppose that we perform a macroscopic momentum balance on the two regions indicated: bulk solution far away from the brush, and at a point within the brush. At equilibrium, the fluid pressure in the bulk and inside the brush must be equal; otherwise, we would get net flux of solvent to rush from one side to the other. Therefore, $p_f^{\mathrm{bulk}} = p_f^{\mathrm{brush}}$. Since the region inside the polymer brush also contains interactions among the polymer beads, these interactions generate an osmotic pressure, Π. This osmotic pressure increases the chemical potential of the solvent in the solution so that it equals that of the excess solvent surrounding the swollen polymer brush. The elastic swelling reaction of the network structure may be interpreted as a pressure acting on the solution, or swollen gel. The chain

stretching and network swelling exactly balances the osmotic pressure generated by their interactions, in such a way that the overall momentum balance is satisfied. Therefore, polymer brush swelling and osmotic pressures are equivalent descriptions of the equilibrium structure of the polymer brush. Once we have a constitutive relation for the polymer, we can directly equate the osmotic pressure with the network swelling to determine the shape and size of the polymer gel. For example, the swelling of a gel is given by Eq. (3.75) in Doi (Soft Matter Physics, 2013) (1):

$$\Pi = G_0 \left(\frac{\phi}{\phi_0}\right)^{1/3}, \tag{1.4}$$

where ϕ is the volume fraction of polymers in the gel (i.e., metric of gel swelling), ϕ_0 is the volume fraction in the reference state, and G_0 is the shear modulus of the gel in the reference state. Paraphrasing Doi's text, the left hand side of Eq. (1.4) represents the force that drives polymers to expand and mix with the solvent, while the right-hand side represents the elastic restoring force of the polymer network which resists expansion. The equilibrium volume of the gel is determined by the balance of these two forces. Once again, this demonstrates that the polymer brush height and osmotic pressures are equivalent descriptions of the equilibrium structure of the polymer brush.

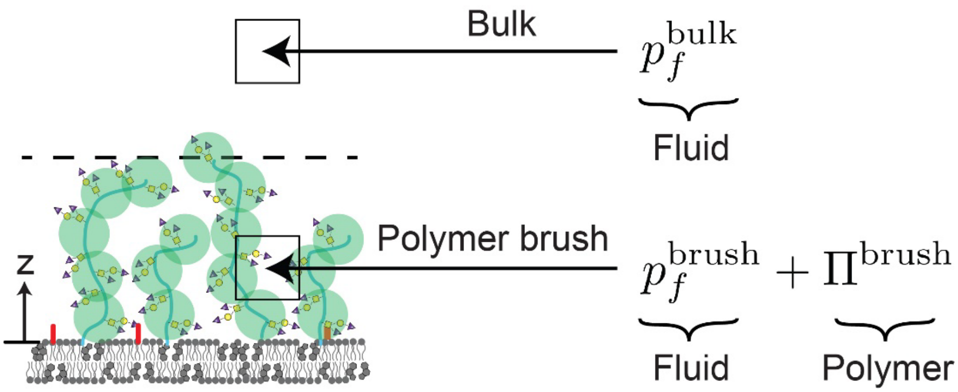

Figure S1.1: Cartoon illustrating the overall suspension pressure, which is composed of both a fluid pressure and the particle (polymer) osmotic pressure. Network swelling and osmotic pressures are equivalent descriptions of the equilibrium structure of the polymer brush.

In the x- and y-directions (tangent to membrane surface), the particle stress can take on any value, $\sigma_{xx}^{(P)} \neq 0$ and $\sigma_{yy}^{(P)} \neq 0$. We therefore have an anisotropic particle stress tensor, $\boldsymbol{\sigma}^{(P)} = \sigma_{xx}^{(P)} \boldsymbol{e}_x \boldsymbol{e}_x + \sigma_{yy}^{(P)} \boldsymbol{e}_y \boldsymbol{e}_y + \sigma_{zz}^{(P)} \boldsymbol{e}_z \boldsymbol{e}_z$, with $\sigma_{zz}^{(P)} = 0$. The mechanical (osmotic) pressure is defined as $\Pi = -\mathrm{tr}\, \boldsymbol{\sigma}/3$, and we define the in-plane pressure of cell surfaces as

$$\Pi = -\left(\sigma_{xx}^{(P)} + \sigma_{yy}^{(P)}\right)/2\,. \tag{1.5}$$

This is the in-plane pressure that we report in this document for both experiments and simulations.

2. Equation of state (EOS) of crowded membrane surfaces

Modeling the cell surface proteins and glycans as coarse-grained polymers, we can obtain an equation of state relating the osmotic pressure of the cell surface suspension as a function of its material properties, including density, contour length, persistence length, and electrostatic charge.

The equation of state for hard-sphere colloidal suspensions, including the Carnahan-Starling EOS, is used commonly to model macromolecular crowding on the membrane surface and inside the cytoplasm (2-

5). Here, we use our data from molecular dynamics (MD) simulations to determine the equation of state for cell surfaces (see Chap. II for details of MD simulations). The EOS may be represented as a virial expansion (6):

$$\Pi = \frac{k_B T}{V_p}\left(\frac{\phi}{N_R} + B_2\phi^2 + B_3\phi^3 + \cdots\right), \tag{1.6}$$

where $V_p = \pi\sigma^3/6$ is the volume of a monomer, $N_R = L/\sigma$ is the degree of polymerization, B_2 and B_3 are the second and third virial coefficients and describe two-body and three-body interactions, respectively. For Weeks-Chandler-Andersen (WCA) potentials between all particles, $B_2 > 0$ and $B_3 > 0$, and the pressure increases beyond the ideal-gas value.

As shown in Fig. S1.2, we find that truncation at the three-body level gives a proficient agreement over the concentrations that we tested in MD. For charge-neutral polymer surfaces, $B_2 = 0.40$, $B_3 = 7.93$. For charged polymers with Debye length $\kappa^{-1} = 1\sigma$, we obtain $B_2 = 2.22$, $B_3 = 10.81$; for $\kappa^{-1} = 2\sigma$, we obtain $B_2 = 2.68$, $B_3 = 29.67$.

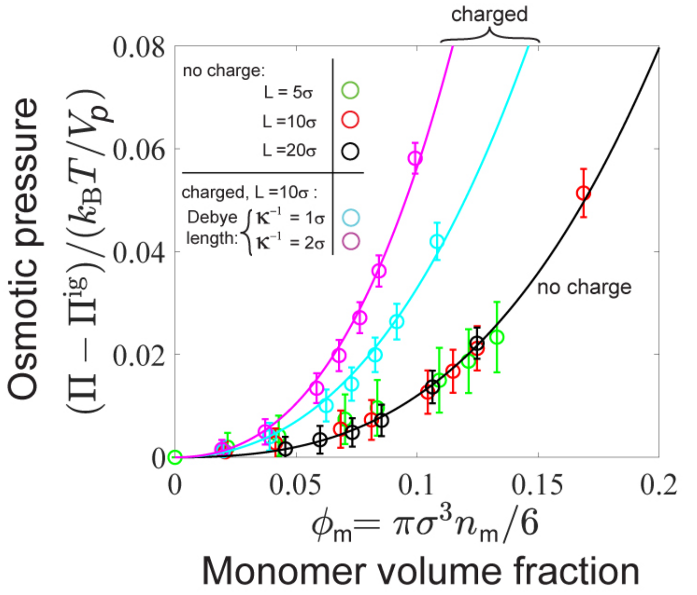

Figure S1.2: Coarse-grained molecular dynamics (MD) simulations were used to compute the osmotic pressure generated by polymers tethered to a membrane surface. The osmotic pressure is defined by Eqs. 1.5 and 1.6, and the ideal-gas pressure has been subtracted in this plot, $\Pi^{\text{ig}} = k_B T\phi/(V_p N_R)$, where V_p is the volume of a monomer and $N_R = L/\sigma$ is the degree of polymerization. The symbols are simulation data, and the solid curves are Eq. 1.6.

3. Analytical theory for brush inclusion penalty

Halerpin et al. (7-9) have conducted theoretical analyses of soluble protein adsorption onto chemically grafted polymer brush surfaces. They distinguish between an insertion versus compressive mechanism of protein adsorption, depending on the size of the protein compared to the mesh size of the polymer brush. One key difference between chemically grafted polymer brushes and the cell surface glycoprotein brush is that a significant fraction of transmembrane proteins is mobile and can translate in 2D along the lipid bilayer surface. Therefore, a large macromolecule may adsorb onto the membrane via the insertion mechanism because it is energetically more favorable to exclude polymers from the interface as opposed to compressing the brush.

To obtain the free energy of inserting a large macromolecule into the cell surface glycocalyx, we follow the theory by Louis et al (10). The inclusion energy of a colloid in a polymer suspension is given by

$$\Delta \mathrm{U} = \Pi(\phi)\mathrm{V_s} + \gamma(\phi)\mathrm{A_s}, \tag{1.7}$$

where $\Pi(\phi)$ is the osmotic pressure, $\gamma(\phi)$ is the interfacial tension, $\mathrm{V_s}$ is the volume of the macromolecule (= $4\pi R^3/3$ for spheres), and $\mathrm{A_s}$ is the surface area of the macromolecule (= $4\pi R^2$ for spheres). The first term in Eq. 1.7 is the reversible work required to create a cavity of volume $\mathrm{V_s}$ within the polymer brush. The second term is the energy penalty associated with creation of a depletion layer around the colloid surface. The osmotic pressure term has been discussed earlier and is given by Eq. 1.6.

The interfacial tension for a planar interface is given by

$$\gamma(\phi) = -\Pi(\phi)\Gamma(\phi) + \int_0^{\phi} \Pi(\phi') \left(\frac{\partial \Gamma(\phi')}{\partial \phi'}\right) d\phi', \tag{1.8}$$

where the relative adsorption $\Gamma(\phi)$ captures the effect of changes in local polymer density due to the creation of an interface. The adsorption is defined as

$$\Gamma(\phi) = \int_0^{\infty} \left(\frac{\phi(r)}{\phi(r\to\infty)} - 1\right) dr, \tag{1.9}$$

where r is the distance from the colloid surface. The leading order expression of the adsorption is approximated by $\Gamma \approx -2R_g/\sqrt{\pi} \approx -R_g$. This assumes that the adsorption is independent of local density and behaves as an ideal polymer. With this approximation, the surface tension is described solely by the entropic free energy penalty of creating an additional cavity of volume $\Gamma \mathrm{A_s} \approx R_g \mathrm{A_s}$, in addition to the volume of the sensor $\mathrm{V_s}$.

Using this approximation, the surface tension term can be incorporated into a bulk osmotic pressure term in the insertion free energy as

$$\Delta U \approx \Pi(\phi) V^{\mathrm{eff}}, \tag{1.10}$$

where the effective volume of the cavity due to macromolecular insertion is $V^{\mathrm{eff}} = 4\pi R^3/3 + 4\pi R^2 R_g$ for a spherical polymer blob of size R_g and macromolecule of size R (see Fig. S1.3). For our system with $R \approx R_g$, the effective volume is well-approximated as $V^{\mathrm{eff}} = 4\pi(R + R_g)^3/3$.

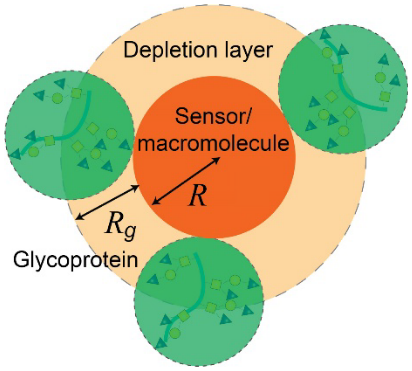

Figure S1.3: Schematic of the depletion layer and cavity related to the inclusion free energy of a colloid inside a polymer brush suspension. The size R is the radius of the macromolecule and R_g is the radius of gyration of the glycoprotein.

We use coarse-grained MD simulations to validate our theory. As shown in Fig. S1.4(A), when the insertion energy is plotted as a function of the surface density of polymers, there is no collapse of the data across different sensor sizes and lengths of the polymer brush. This indicates that the surface density is not an accurate, quantitative metric of crowding. Other proxy metrics like molecular weight and number density are inadequate to characterize crowding, because they do not include effects of variable glycoprotein length, polydispersity, stiffness, density, charge, and other interactions.

However, when the energy is plotted as a function of the osmotic pressure, the data collapse onto a master curve, as shown in Fig. S1.4(B). It is important to note that there is no adjustable fitting parameters here. These results confirm our hypothesis that the surface osmotic pressure is the unique quantity that provides a direct, quantitative metric of crowding. When quantifying crowding effects on cell surfaces, one should use the osmotic pressure instead of other proxy metrics like molecular weight, size, and surface density of the surface proteins.

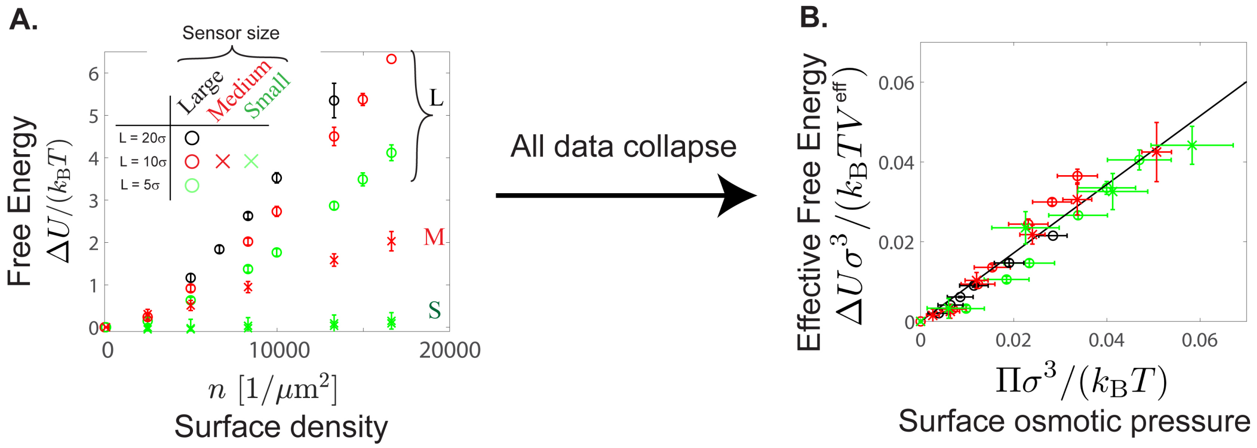

Figure S1.4: Insertion energy for large ($R = 2\sigma$), medium ($R = \sigma$), and small ($R = \sigma/2$) sensors as a function of surface polymer number density and contour lengths. (A) Insertion energy for the sensors as a function of surface density of polymers. The data do not collapse onto a universal curve, indicating that the surface density is not an accurate metric of surface crowding. (B) Normalized insertion energy, $\Delta U\sigma/(k_B T V^{\text{eff}})$, where $V^{\text{eff}} = 4\pi(R + R_g)^3/3$ is the excluded volume of the sensor in the polymer brush. Data across different sensor sizes and surface polymer contour lengths collapse onto a universal line when plotted as a function of osmotic pressure Π.

4. Analytical theory for effective brush potential

In our coarse-grained MD simulations, the Weeks-Chandler-Andersen (WCA) repulsive potential (11) was implemented between the polymer-polymer, polymer-sensor, polymer-substrate, and sensor-substrate. For the sensor-substrate interactions, we also add an attractive Morse potential to model affinity of the sensor to the membrane, which effectively approximates a short-range, attractive harmonic potential, $k(z-r_0)^2/2$, with stiffness k. These two potentials are sufficient to predict the free-energy landscape for the bare membrane simulations.

$$U_0 = \begin{cases} U_{\text{WCA}} + U_{\text{att}} & \text{if } z < 2^{1/6}R \quad (1.11) \\ U_{\text{att}} & \text{if } 2^{1/6}R < z < (2^{1/6}R + R) \quad (1.12) \\ 0 & \text{if } z > (2^{1/6}R + R) \quad (1.13) \end{cases}$$

where the repulsive WCA potential is given by

$$U_{\mathrm{WCA}} = 4\epsilon\left[\left(\frac{R}{z}\right)^{12} - \left(\frac{R}{z}\right)^{6}\right] + \epsilon \tag{1.14}$$

and the attractive potential to the membrane surface is given by

$$U_{\mathrm{att}} = k(z-R)^2/2 \ . \tag{1.15}$$

Obtaining the ΔU contribution to the overall free energy is non-trivial, since we only include WCA potentials for sensor-polymer interactions and no explicit potential of the brush into the sensor's equation of motion. We do not apriori know what the effective potential posed by the polymer brush will be.

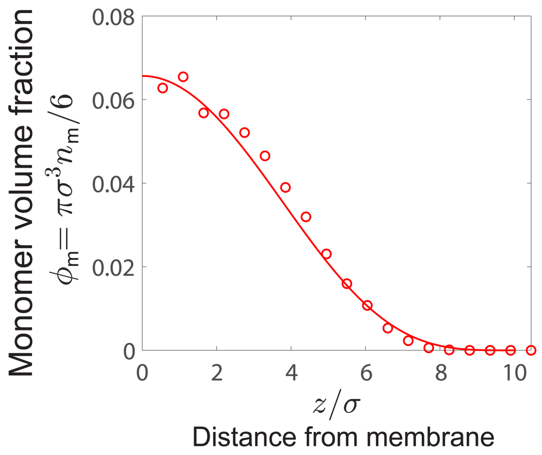

Figure S1.5: Local monomer volume fraction as a function of distance from the membrane, for a surface density of 8000 chains/μm^2. The symbols are simulation data, and the solid curve is Eq. 1.16.

From MD simulation data, the monomer volume fraction of the polymers as a function of height from the membrane surface is modeled by

$$\phi_m = \phi_m^0\left[1 - \left(\frac{z}{L}\right)^2\right]^4 . \tag{1.16}$$

We verified that this model for the polymer brush agrees with data from MD simulations, as shown in Fig. S1.5. The parabolic form inside the brackets is well known from polymer theory (12); we observe a steeper decay of density, likely due to the fact that the chains are not grafted and instead free to diffuse in 2D. The free energy of a sensor binding to a crowded polymer surface is given by

$$U = \begin{cases} U_{\mathrm{WCA}} + U_{\mathrm{att}} + \Delta U & \text{if } z < 2^{1/6}R & (1.17) \\ U_{\mathrm{att}} + \Delta U & \text{if } 2^{1/6}R < z < (2^{1/6}R + R) & (1.18) \\ \Delta U & \text{if } z > (2^{1/6}R + R) & (1.19) \\ 0 & \text{if } z > L & (1.20) \end{cases}$$

Substituting Eq. 1.16 into the virial pressure equation of state, Eq. 1.6, gives the local osmotic pressure as a function of distance from the membrane surface. The crowding energy as a function of distance, $\Delta U(z)$, is obtained by using Eq. 1.10. The resulting crowding energy is used in Eqs. 1.17 – 1.20 to obtain the total energy as a function of distance, $U(z)$. As shown in Fig. S1.6, the data from our MD simulations (black symbols) demonstrate excellent agreement with our theory (red curve).

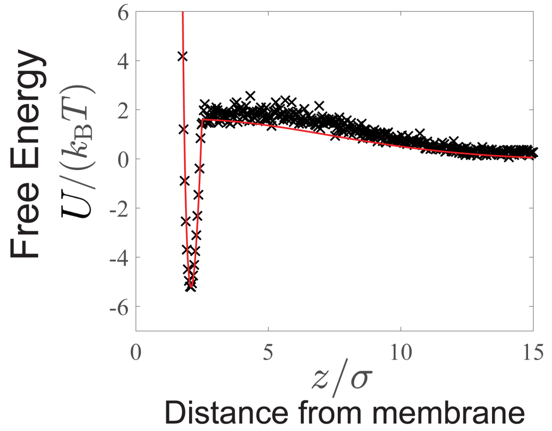

Figure S1.6: Full free energy curve using Eqs. 1.17 - 1.20 (solid red curve) agrees with the direct calculation from the sensor probability distribution (black crosses). There are no adjustable parameters in the theoretical model, Eqs. 1.17 – 1.20.

Chapter II. Coarse-grained molecular dynamics (MD) simulations

1. Basic construction of the model

To construct a molecular model of macromolecular transport across cell surface proteins and glycocalyx, we performed coarse-grained Molecular Dynamics (MD) simulations of colloidal transport within semi-flexible polymers diffusing on 2D surfaces. This is a modified extension of our protein polymer surface model presented previously (13).

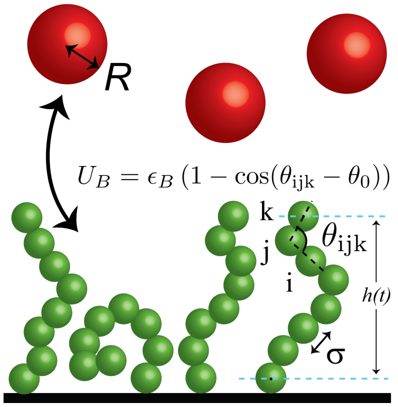

Figure S2.1: Schematic of our coarse-grained MD simulations of a protein polymer brush with surface-binding macromolecular sensors. Using a Kremer-Grest bead-spring model (14), individual beads of diameter σ are connected by a finitely extensible nonlinear elastic (FENE) potential and a bending stiffness is invoked by a 3-particle angular potential, U_B. The bottom bead is confined to remain along a 2D surface but can diffuse laterally along the surface. Sensor particles of size R are added to the bulk above the polymer brush. The sensors have a short-ranged binding affinity to the substrate.

We modeled surface protein chains using a Kremer-Grest bead-spring model (14), with each bead representing a structured protein domain or a coarse-grained unit of an intrinsically disordered domain. A bead at one end of the chain is confined between two parallel walls separated by one bead diameter, allowing it to diffuse freely in 2D but cannot escape out of plane. All other beads on the chain are free to move in

3D, except through a bottom wall that acts as a solid substrate, thereby modeling protein diffusion along a membrane that is in-plane fluid. Because the size of each protein domain is large compared to the surrounding solvent molecules, the solvent is coarse grained and its dynamics are not explicitly evolved. In other words, the protein chain experiences a hydrodynamic drag and Brownian motion from the continuous solvent. In this work, the membrane does not deform nor fluctuate out of plane, although these effects may be included.

Simulations were performed using a GPU-enabled HOOMD-blue molecular dynamics package (15,16), and all simulations contained at least 2000 protein chains and 10000 sensor particles. The dynamics of particle i is evolved in time following the overdamped Langevin equation

$$\mathbf{0} = -\zeta_i \boldsymbol{U}_i + \boldsymbol{F}_i^B + \boldsymbol{F}_i^P, \tag{2.1}$$

where ζ_i is the hydrodynamic drag factor, $\boldsymbol{U}_i$ is the velocity, $\boldsymbol{F}_i^B = \sqrt{2\zeta_i^2 D_i \boldsymbol{\Lambda}}$ is the translational Brownian force, D_i is the Stokes-Einstein-Sutherland translational diffusivity of a single monomer, and $\boldsymbol{F}_i^P$ is the interparticle force. The left-hand side is zero since inertia is negligible for coarse-grained proteins embedded in a viscous solvent. The translational diffusivity is modeled with the usual white noise statistics, $\langle \boldsymbol{\Lambda}(t) \rangle = \mathbf{0}$ and $\langle \boldsymbol{\Lambda}(t)\boldsymbol{\Lambda}(0) \rangle = \delta(t)\, \boldsymbol{I}$, where $\delta(t)$ is a delta function and $\boldsymbol{I}$ is the identity tensor. The drag coefficient ζ_i of particle i is linearly scaled with the particle size σ_i. The interparticle force (described below) includes contributions from sensor-polymer chain interactions, intrachain polymer potential, interchain bead-bead pair interactions, and bead-wall interactions.

All interactions between the particles and walls are modeled with a Weeks-Chandler-Andersen (WCA) potential (11), in which a Lennard-Jones (LJ) potential is shifted upwards, truncated at the potential minimum of $2^{1/6}\,\sigma_i$ (such that the potential is purely repulsive), and assigned a well depth of $\epsilon = k_B T$ (where $k_B T$ is the thermal energy). All lengths are expressed in units of the LJ diameter σ and is set to unity. We connect the polymer chains with a finitely extensible nonlinear elastic (FENE) potential, using a spring constant of $k_0 = 30$ and a bond length of $R_0 = 1.5$ (expressed in terms of reduced LJ units, $\epsilon = \sigma = 1$). To model semi-flexible polymers, we implemented a bending potential between 3 neighboring particles to capture chain stiffness, $U_B = \epsilon_B\big(1 - \cos(\theta_{\text{ijk}} - \theta_0)\big)$, where U_B is the bending energy, θ_{ijk} is the bond angle between neighboring particles (i,j,k), and $\theta_0 = \pi$ is the resting angle. The persistence length of the chains was measured by calculating the bond angle correlation, $\langle \boldsymbol{e}_1 \cdot \boldsymbol{e}_i \rangle = \exp(-s_i/\ell_P)$, where $\boldsymbol{e}_i$ is the unit vector connecting the center of mass positions of particles i and $i + 1$, and s_i is the path length along the polymer to particle i. To implement the persistence length ℓ_P as an input to the simulations, the bending energy is set to $\epsilon_B/(k_B T) = \ell_P/\sigma$ to achieve the desired polymer stiffness. We have set $\ell_P = 3\sigma$ for all of our protein-based simulations based on previous work, and have verified the angular correlation for unbound polymers in 3D as a validation of proper implementation (13).

Sensor particles of different sizes were added to the bulk of the simulation box to model the dynamics of sensor transport and binding to the cell surface. The diameters of the Chol-0.5k PEG, 10k dextran, and 40k dextran sensors were modeled with spheres of diameter 3, 5, and 10 nm, respectively (17). The sensor particles have an attractive potential to the membrane via a Morse potential with the HOOMD parameters ($D_0 = 3 \times 10^9$, $\alpha = 10^{-4}$, $r_0 = \sigma/2$, and $r_{\text{cut}} = \sigma/2 + R$), which effectively approximates a short-range, attractive harmonic potential $U_{\text{att}} = k(z - r_0)^2/2$ with stiffness $k = 2D_0\alpha^2 = 60$. All other particle pairs experience a short-ranged repulsive WCA potential, as described earlier. The dynamics of sensor particle i is evolved in time following the Langevin equation

$$\mathbf{0} = -\zeta_i \boldsymbol{U}_i + \boldsymbol{F}_i^B + \boldsymbol{F}_i^P + \boldsymbol{F}_i^{\text{wall}}, \tag{2.2}$$

where $\boldsymbol{F}_i^{\text{wall}} = \boldsymbol{F}_i^{\text{att}} + \boldsymbol{F}_i^{\text{WCA}}$ is the short-ranged potential that the sensor particle experiences with the surface, $\boldsymbol{F}_i^{\text{att}} = -\nabla U_{\text{att}}$ is the attractive linear force, and $\boldsymbol{F}_i^{\text{WCA}} = -\nabla U_{\text{WCA}}$ is the WCA potential with the surface (see Eqs. 1.14 and 1.15 for definitions of the potentials). In summary, each sensor particle experiences the potentials in Eqs. 1.11 – 1.13 as a function of height z, in addition to the pair-wise WCA potentials with the

protein polymers. It is straightforward to add flexibility and non-spherical shape effects into our sensor model.

Unlike our previous work (13) where a large concentration of passive PEG depletants were added to the bulk to compress the cell surface proteins, in this work we focus on dilute concentrations in which the osmotic compression is negligible. Furthermore, we did not observe any depletion flocculation nor any large-scale protein clustering. Simulations with dilute concentrations of sensors are very time consuming because acquiring sufficient statistics requires many time steps at equilibrium. Therefore, we accelerated our simulations by using a larger concentration of sensor particles that interact only with the protein polymer and the surface, but not with each other. The sensors act as "ideal-gas" particles to each other but interact with WCA potentials with the surface proteins. However, sensor concentrations that are too large can interact with each other indirectly via correlated motion of the protein polymers. A sensor volume fraction less than 1% was used in this work.

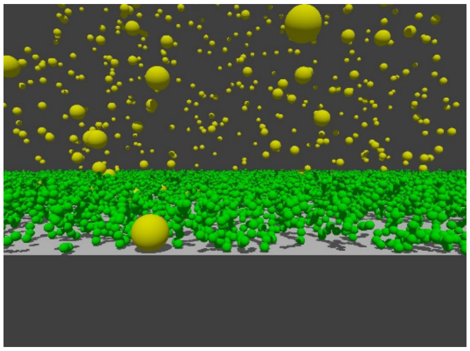

Figure S2.2: Snapshot of coarse-grained MD simulations with sensors (yellow particles) of size $R = 2\sigma$, and surface polymers (green particles) of length $L = 10\sigma$, where σ is the size of an individual monomer.

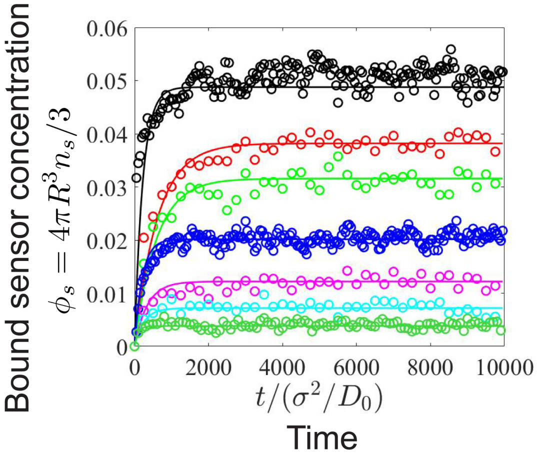

Figure S2.3: Volume fraction of sensors of size $R = \sigma$ on the membrane surface, $\phi_s = 4\pi R^3 n_s/3$, as a function of nondimensional time $t/(\sigma^2/D_0)$, where D_0 is the Stokes-Einstein-Sutherland diffusivity based on the monomer size σ. Solid curves are a fit to a first-order kinetic rate law, $\phi_s/\phi_s^\infty = 1 - \exp(-t/\tau)$.

We used a system box size of $V = L^2 L_z$, where L was adjusted to achieve the specified area density and number of polymer chains, and L_z was chosen to be sufficiently large to maintain a uniform bath concentration of sensors in the bulk. Dilute surface densities were conducted with ~ 200 chains/μm^2, and the dense surfaces contained ~ 20,000 chains/μm^2. We imposed periodic boundary conditions in the x and y directions, and no-flux hard walls at $z = 0$ and $z = L_z$ to prevent any particles from passing through. Initial configurations were generated by placing the particles in lattice locations, and sufficient time steps were run to reach a steady-state. Time steps were varied from $\Delta t = 10^{-6} - 10^{-4}$ and verified to be sufficiently small to capture relevant dynamics.

A snapshot of the simulations is shown in Fig. S2.2. Simulations were conducted for long enough time steps to sample equilibrium properties. Figure S2.3 shows the concentration of sensors on the membrane surface as a function of time, and we can see that sufficient configurations are sampled after equilibrium is achieved.

2. Calculation of mechanical stress (and pressure) of cell surface proteins

The pressure of cell surface proteins is given by the negative trace of the virial stress tensor,

$$\boldsymbol{\sigma} = -nk_BT\boldsymbol{I} - \frac{1}{V}\langle \sum_i \sum_{j>i} \boldsymbol{r}_{ij}\boldsymbol{F}_{ij}^P \rangle, \tag{2.3}$$

where $V = L^2\langle h \rangle$ is the volume of the polymer brush, L is the length of the simulation box, $\langle h \rangle$ is the average height of the polymer surface, $\boldsymbol{r}_{ij}$ is the interparticle distance, and $\boldsymbol{F}_{ij}^P$ is the pair-wise interparticle force between all particles, including individual monomers within a single polymer. The first term in Eq. 2.3 is the ideal-gas stress and the number density is defined as $n = N/V$ where N is the total number of particles. The second term is the Irving-Kirkwood virial stress tensor that measures the contribution from interparticle interactions. For polymers, it is important to note that the ideal-gas pressure is $\Pi^{\mathrm{ig}} = n_c k_\mathrm{B}T$ and not $nk_\mathrm{B}T$, where n_c is the number density of chains, $n_c = N_c/V$ (N_c is the total number of chains). Particles lose their translational degree of freedom when constrained as a polymer, so the ideal-gas pressure decreases with increasing degree of polymerization. Returning to our virial EOS in Eq. 1.6, we can rewrite the ideal-gas expression as $\Pi^{\mathrm{ig}} = (k_\mathrm{B}T/V_p)\phi/N_R$, where the degree of polymerization $N_R = N/N_c$. For very large molecular weight polymers ($N_R \to \infty$), the ideal-gas contribution is zero and the first term contributing to the osmotic pressure is two-body interactions (i.e., second virial coefficient).

In calculating the osmotic pressure using Eq. 2.3, it would seem like we are overestimating the pressure based on the first term, which assumes that all N particles are independent. However, the virial stress term will cancel the "excess" stress contribution from the $nk_\mathrm{B}T$ term. That is, the FENE potential applied to the constitutive particles on the polymers (which represent elasticity) has the opposite sign and subtracts the independent degrees of freedom. The net result is that we correctly recover the ideal-gas pressure of a polymer, $\Pi^{\mathrm{ig}} = (k_\mathrm{B}T/V_p)\varphi/N_R$.

3. Calculation of free energy potentials

The free energy experienced by the sensor particle as a function of the distance from the membrane surface is given by $U = -k_\mathrm{B}T \ln P(z)$, where $P(z)$ is the normalized probability distribution of the sensors. We calculate $P(z)$ by binning the simulation into thin slabs and ensemble averaging over all particles and time. From these distributions, we calculate the effective binding energy and the insertion penalty at equilibrium, as shown in Fig. S2.4. The offset at the energy minimum gives the glycocalyx contribution U_g and the in-plane osmotic pressure of the cell surface.

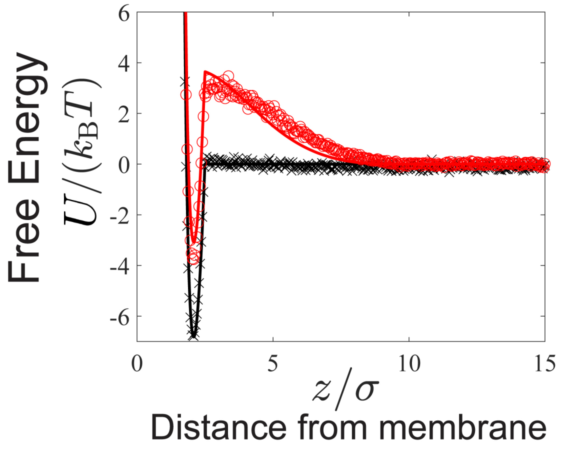

Figure S2.4: Effective free energy potential experienced by a macromolecule of size $R = \sigma$ approaching a bare (black) and crowded (red) cell surface membrane, which is a superposition of two contributions, U_0 and ΔU. The circle and cross symbols are a direct calculation of the free energy via $U = -k_\mathrm{B}T \ln P(z)$ from MD simulations, where $P(z)$ is the normalized probability distribution of the sensors. Solid curves are Eqs. 1.17-1.20. The depth of the minimum gives the effective binding energy at equilibrium.

As shown in Fig. S2.4, our analytical theory in Eqs. 1.17-1.20 agrees very well with the direct energy calculations from MD simulations. Once the sensor-substrate affinity is determined in the theory by fitting to the bare-membrane simulations (black symbols and curve), there are no fitting parameters for the crowded surface simulations and theory (red symbols and curve).

4. Incorporation of charges on protein polymers

Charge effects were analyzed in MD simulations by adding a screened coulomb (Yukawa) pair potential between the constituent monomers of the polymer. The energy scale was set to $\epsilon = k_{\mathrm{B}}T$, and the Debye length was varied from $\kappa^{-1} = \sigma$ to $\kappa^{-1} = 2\sigma$. Electrostatic interactions were not included for sensor-polymer or sensor-sensor interactions.

Chapter III. Experimental details

1. Binding isotherms for dextran sensors

In the main text, we obtained the relative difference between the crowded and bare surfaces by taking the ratio of the slopes of the bound sensors at small bulk sensor concentrations. This provides a direct measurement of the relative difference, K_D/K_D^0 (or K_D/K_D^{proK} for RBCs), assuming that the bound saturation concentration, C_{max}, is equal on the crowded and bare surfaces. As a control, we performed a measurement of the effective dissociation constant of the dextran sensors by obtaining the full isotherm across a larger range of bulk concentrations. The maximum saturating concentrations of the sensors, C_{max}, were obtained by incubating the beads and RBCs with 0.5 μM bulk sensor concentration. The bound sensor concentrations at each bulk sensor concentrations were normalized by the maximum concentration at saturation to obtain the fractional surface coverage, $\theta = C/C_{\mathrm{max}}$. The slope at small bulk concentrations were used to obtain the dissociation constant. A representative dataset containing the full isotherm on RBCs for the dextran 40k sensor is shown in Fig. S3.1. The relative ratios of the binding affinities were $K_D/K_D^{\mathrm{proK}} = 1.5$ and 2.6 for sialidase-treated and untreated RBCs, respectively, which agree with the results in the main text obtained using a ratio of the slopes at small bulk concentrations.

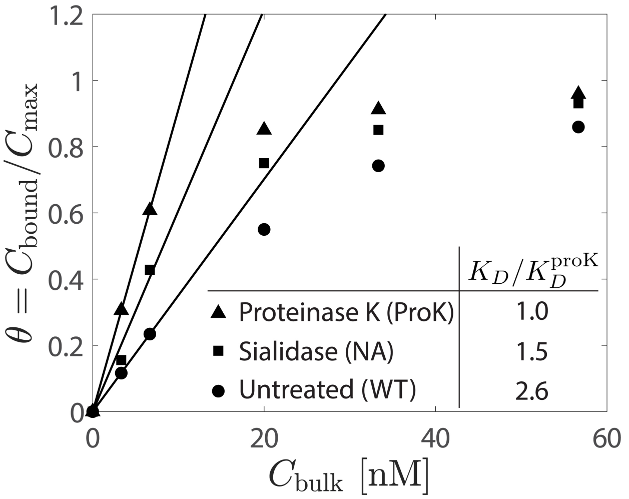

Figure S3.1. Surface coverage of bound 40k dextran sensors as a function of bulk concentration of sensors on untreated (circle), sialidase (NA)-treated (square), and Proteinase K-treated (triangle) RBCs. Each measurement was normalized by the maximum concentration at saturation to obtain the fractional surface coverage, $\theta = C/C_{\mathrm{max}}$. The slope at small bulk concentrations, shown as solid lines, were used to obtain the dissociation constant.

2. Effective dissociation constants of multivalent binding species

As shown by Cremer et al. (18,19), interpretation of the effective dissociation constant of multivalent binding species requires some care. For bivalent-binding antibodies, there are two dissociation constants, K_{D1} and K_{D2}, defined by $K_{D1} = [A][L]_s/[AL]_s$ for the first binding event, and $K_{D2} = [AL]_s[L]_s/[AL_2]_s$ for the second binding when both binding sites are bound to the two ligands. The total surface concentration of sites is $[L]_s/2 + [AL]_s/2 + [AL_2]_s$, so the fraction of bound species is

$$\theta = \frac{\text{total bound antibodies}}{\text{total number of sites}} = \frac{[AL]_s + [AL_2]_s}{[L]_s/2 + [AL]_s/2 + [AL_2]_s} = \frac{\alpha[A]}{K_D + [A]}$$

where $\alpha = (K_{D2} + [L]_s)/(K_{D2} + 2[L]_s)$ is a number that varies between 0.5 and 1.0, and the effective dissociation constant is given by

$$K_D = \frac{K_{D1}K_{D2}}{K_{D2} + 2[L]_s}$$

Therefore, the binding isotherm still takes on the classic Langmuir form, except the interpretation of the effective K_D is slightly different. At small bulk antibody concentrations, $[A] \ll 1$, we are still fitting a line with the slope that gives the effective dissociation constant. The main potential issue is that the effective dissociation constant is now a function of the ligand concentration, $[L]_s$. This is not an issue for our dextran-based sensors because the "ligand" concentration is simply the total available surface area on the beads and cells, which is fixed constant in each respective experiment.

However, this is a potential issue for our antibody-based measurement because our biotin-DNA cholesterol sensors incorporate into cell membranes with different affinities. Therefore, the ligand concentration $[L]_s$ could be changing drastically as a function of cell type, over-expression, etc. As shown in the expressions above, varying $[L]_s$ will change the effective dissociation constants, which is an unwanted problem. In fact, we did indeed see this effect when we used a very large concentration of the biotin-based cholesterol sensors.

This issue has been addressed previously by Yang et al (19), in which they observed a 10x decrease in effective K_D of monoclonal antibodies on SLB-coated beads when the ligand surface density was increased from $1400/\mu m^2$ to $40{,}000/\mu m^2$. Fortunately, they found that the effective K_D increased only from $13\mu M$ to $18\mu M$ when increasing the ligand surface density from $1400/\mu m^2$ to $4200/\mu m^2$ (small ligand concentrations). We therefore hypothesized that staying at small surface ligand concentrations would reduce or eliminate this potential uncertainty of determining the effective K_D.

To these ends, we performed control experiments with varying amounts of biotin-based cholesterol sensors on beads, RBCs, and mammalian cells. We found that the effective dissociation constant was insensitive to changes in $[L]_s$ when $[L]_s \ll 1$. In other words, the parameters α and K_D above were not a function of $[L]_s$ at the low ligand concentrations used in our measurements.

The rationale for this result in the context of the equations above is that $\lim_{[L]_s \to 0} \alpha \to 1$ and $\lim_{[L]_s \to 0} K_D \to K_{D1}$. In other words, we are measuring essentially the monomeric binding affinity of the antibody because the surface ligand concentration is so low, and the second binding constant K_{D2} drops out of the problem. Therefore, our approach to obtain the effective dissociation constant from the Langmuir isotherm form is an appropriate way to obtain the binding affinities and energies, and our results are insensitive to the absolute surface concentrations of the ligand sensors.

3. Sensors have no non-specific adsorption onto membranes

Our dextran sensors have a strong affinity to insert into the lipid bilayer via cholesterol tags bound to the dextran molecules. To rule out the possibility of other, non-specific interactions of the dextran sensors onto membranes, we conjugated the dextran molecules to Alexa Fluor dyes only, without cholesterol tags. Upon incubating these dextran-dye sensors with lipid-coated beads and red blood cells (RBCs), we did not detect

enrichment on the membrane, validating that the main adsorption mechanism of our sensors is via the cholesterol tags. A representative microscopy image is shown in Fig. S3.2. We conclude that there is a negligibly small adsorption and non-specific interaction of our dextran macromolecules with the membrane. For our other probe based on anti-biotin binding, an antibody-only control was performed on cholesterol anchors containing no biotin tag (a cholesterol-FITC construct), and we observed no nonspecific anti-biotin binding on the membrane.

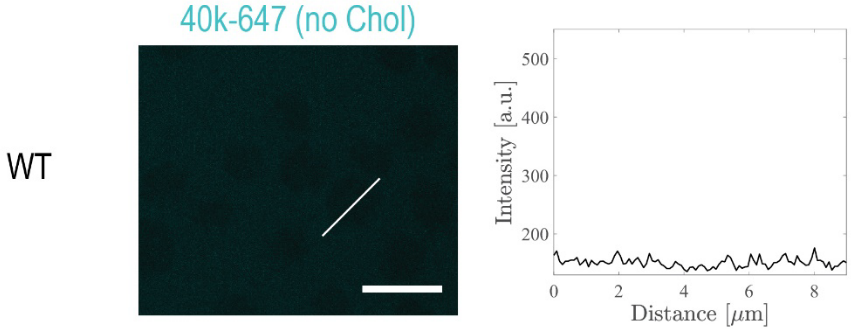

Figure S3.2. There is no non-specific adsorption of our sensors onto membranes. Dextran-dye sensors in the absence of cholesterol does not incorporate into lipid coated beads or RBC membranes.

4. Control experiments of dextran sensors with different chemistries.

In the previous section, we showed that the intrinsic dextran macromolecules (without cholesterol) do not exhibit non-specific adsorption onto the membrane. However, the Alexa Fluor dyes conjugated to the cholesterol-dextran macromolecules are negatively charged and may interact electrostatically with the charged cell surface. As a control, we conjugated our cholesterol-dextran sensors with BODIPY, a charge-neutral dye. As shown in the main text, RBCs have highly charged cell surfaces that we can modulate using neuraminidase (NA) treatment; RBCs are a good model system to examine the interactions between the cell surface and the sensor dye charges.

We conducted two tests on RBCs to assess the impact of the dye charges. First, to test whether the binding kinetics are equivalent between the Alexa Fluor and BODIPY conjugated cholesterol-dextran sensors, we took a time series of sensor binding on untreated and neuraminidase (NA) treated RBCs. As shown in Fig. S3.3, the binding kinetics of both dye types are essentially identical across the RBC surfaces. Note that the important feature from Fig. S3.3 is the time constant towards equilibrium, not the final saturation value. The final equilibrium value is irrelevant because the intensity units from the flow cytometer is different across the two different laser channels, and because the two sensor types may contain a slightly different labeling ratio of cholesterol. The dashed and solid curves in Fig. S3.3 are a fit to a first-order kinetic rate law, $C/C_\infty = 1 - \exp(-t/\tau)$. The characteristic decay time towards equilibrium is $\tau = 7.53$ min (WT) and $\tau = 7.05$ min (NA) for the Alexa Fluor 647 conjugated dextran sensors and $\tau = 7.38$ min (WT) and $\tau = 7.51$ min (NA) for the BODIPY conjugated dextran sensors. All of these values are similar to each other, which validates that the binding kinetics between the charged Alexa Fluor dyes and neutral BODIPY dyes have no difference. Within the same dye, the fact that the binding kinetics are the same between the WT cells (fully charged) and NA-treated cells (reduced charge) is another indication that the kinetics are unaffected by cell surface charges.

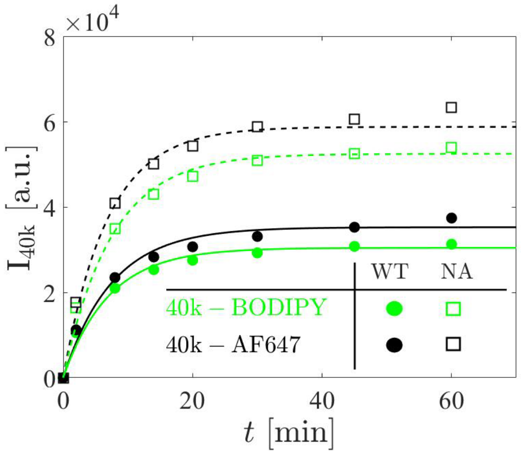

Figure S3.3. Bound sensor concentration as a function of time for Alexa Fluor 647 conjugated dextran sensors (black curves and symbols) and BODIPY conjugated dextran sensors (green curves and symbols), on untreated RBCs ("WT", filled circle symbols) and NA-treated RBCs ("NA", open square symbols). Solid and dashed curves are a fit to a first-order kinetic rate law, C/C_∞ = 1 - exp(-t/τ). The characteristic decay time towards equilibrium is τ = 7.53 min (WT) and τ = 7.05 min (NA) for the Alexa Fluor 647 conjugated dextran sensors and τ = 7.38 min (WT) and τ = 7.51 min (NA) for the BODIPY conjugated dextran sensors. This validates that the binding kinetics between the charged Alexa Fluor dyes and neutral BODIPY dyes has no difference.

Next, we tested whether the effective dissociation constant K_D is different between the Alexa Fluor and BODIPY conjugated cholesterol-dextran sensors. As described in the main text, we performed an equilibrium measurement of sensor binding on untreated and NA-treated RBCs using both types of sensors. The binding measurement at large bulk concentrations provide the saturating binding concentration, C_max, and the fractional coverage may be obtained using small bulk concentrations, $\theta = C/C_\mathrm{max}$. As presented in the main text, our key metric of comparison is the relative difference between the binding affinities of WT and NA-treated cells, not the absolute value of K_D. As shown in Fig. S3.4, we find that the ratio of the binding affinities on NA-treated and WT RBCs are $K_\mathrm{D}^{NA}/K_\mathrm{D}^{WT} = 0.634$ for BODIPY conjugated cholesterol-dextran and $K_\mathrm{D}^{NA}/K_\mathrm{D}^{WT} = 0.652$ for Alexa Fluor 647 conjugated cholesterol-dextran. This validates that the negative charges on the Alexa Fluor 647 dyes do not impact the results presented in Fig. 2 of the main text.

We believe that the negative charges on the dextran sensors play a small role for our particular system in part because the bound sensor concentrations are very dilute on the RBC surface. The average separation distance between the dextran sensors on the RBC surface over the range of interest is $\approx$ 100 nm at $100/\mu m^2$ surface density, and the average distance between the dextran sensors and the GYPA proteins is $\approx$ 10 nm at $2000/\mu m^2$ GYPA surface density. Both of these distances are more than an order of magnitude larger than the small 0.7 nm Debye length in physiological buffers, so we expect that the majority of the charge interactions are screened. Interestingly, the absolute magnitudes (not the ratio) of K_D for the BODIPY sensors are larger than the Alexa Fluor sensors, which is the opposite of what one may expect based on electrostatic repulsion between the dye and the charged cell surface. One may naively expect that the negative charge repulsion between the Alexa Fluor and the RBC surface would cause less binding and a larger K_D for the Alexa Fluor sensors. At very large sensor concentrations, the surface is saturated with a dense packing of sensors. Here, we believe that the average separation distance between neighboring sensors may be similar to the Debye length, and the negative charge repulsion among the neighboring sensors' Alexa Fluor dyes may lead to smaller packing and a smaller C_max than the BODIPY conjugated dextran sensors. A smaller saturating concentration would normalize the fractional coverage to a larger value, leading to smaller absolute magnitude of K_D for the Alex Fluor conjugated dextran sensors. In summary, the effects of negative charges on the dye do not impact the normalized ratio of the binding affinities at the dilute concentrations that form the basis of our crowding measurements; however, they are

an important consideration in general when the absolute magnitudes of K_D are desired or one would like to use these sensors at much larger concentrations.

Once again, the absolute magnitudes of K_D are unimportant for our work because we are only interested in relative ratios between the different cell treatments. The crowding free energies come from the ratio in the binding affinity on bare and crowded surfaces, $\Delta U = k_B T \ln(K_D/K_D^0)$, not the absolute magnitude. The absolute magnitudes for the dextran sensors depend on the intrinsic chemistries of the cholesterol interaction with the membrane, which is irrelevant for our crowding measurements.

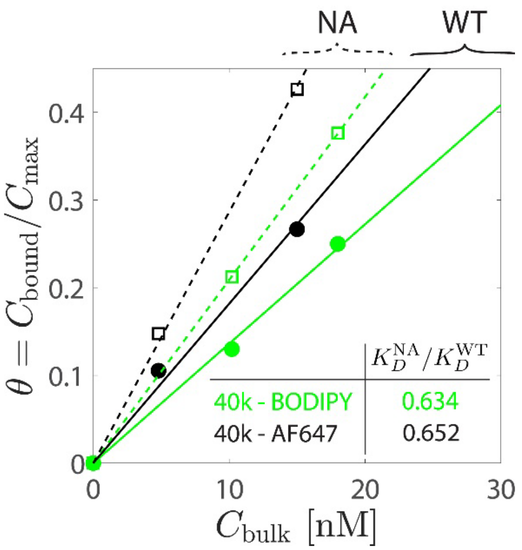

Figure S3.4. Surface coverage of bound 40k dextran sensors as a function of bulk concentration of Alexa Fluor 647 conjugated dextran sensors (black lines and symbols) and BODIPY conjugated dextran sensors (green lines and symbols), on untreated RBCs ("WT", filled circle symbols) and NA-treated RBCs ("NA", open square symbols). Solid and dashed lines are a fit to obtain the dissociation constant. We find that the ratio of the binding affinities on NA-treated and WT RBCs are $K_\mathrm{D}^{NA}/K_\mathrm{D}^{WT} = 0.634$ for BODIPY conjugated cholesterol-dextran and $K_\mathrm{D}^{NA}/K_\mathrm{D}^{WT} = 0.652$ for Alexa Fluor 647 conjugated cholesterol-dextran. This validates that the negative charges on the Alexa Fluor 647 dyes do not impact the results presented in Fig. 2 of the main text.

5. Verification that the stock sialidase is protease-free.

A sodium dodecyl sulfate polyacrylamide gel electrophoresis (SDS-PAGE) protein gel of bovine serum albumin (BSA), MW = 66 kDa, is shown in Fig. S3.5. BSA was treated with various concentrations of sialidase and Proteinase K at 37C for 5 hours. The protein was subsequently heat-denatured in 1x Laemmli Sample Buffer (Sigma Aldrich) in the presence of β-mercaptoethanol. The sample was then loaded onto a NuPAGE Novex 4–12% gradient Bis-Tris gel (Fisher Scientific) and separated by electrophoresis. This gel confirms that our stock sialidase does not have protease activity. In addition to BSA, we collected the supernatant of RBCs treated with sialidase and found no detectable signal of proteins released from the RBC surface as a result of sialidase treatment. This verifies that soluble sialic acid-binding proteins are not embedded in the glycocalyx to hinder the binding of the sensors.

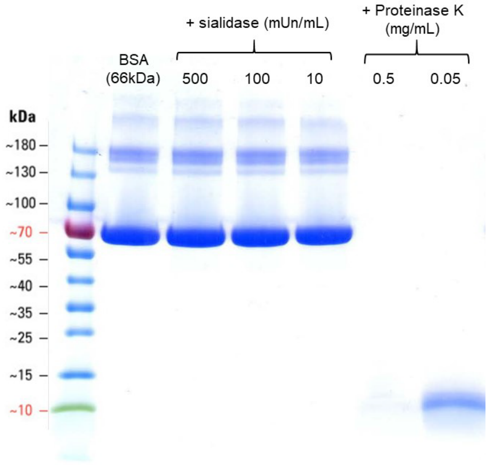

Figure S3.5. Sodium dodecyl sulfate polyacrylamide gel electrophoresis (SDS-PAGE) protein gel of bovine serum albumin (BSA), MW = 66 kDa, treated with enzymes sialidase and Proteinase K at 37C for 5 hours. We detected no protease activity in our stock sialidase. In our experiments, RBCs were treated with sialidase at 50 mUn/mL.

6. EDC conjugation to remove negative charges from cell surface

We used a carbodiimide crosslinker chemistry based on 1-Ethyl-3-(3-dimethylaminopropyl)carbodiimide (EDC) to remove negative charges present on carboxylic acid groups on the red blood cell surface. Most surface negative charges come from the carboxylic acid on the sialic acids, but charged amino acids (e.g., aspartic acid and glutamic acid) may also be impacted by this reaction. We added EDC and hydrazide-biotin (a charge-neutral molecule) to a suspension of red blood cells, and the effectiveness of the reaction was assessed by imaging with AF555-labeled straptavidin. As shown in Fig. S3.6, the reaction was qualitatively very effective and we believe that most negative charges were neutralized on the cell surface.

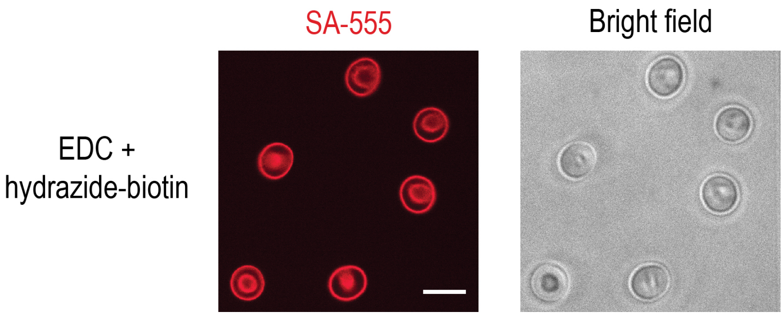

Figure S3.6. EDC chemistry was used to remove negative charges from the red blood cell surface. The reaction conjugated hydrazide-biotin to carboxylic acid sites, which enabled detection using AF555-labeled streptavidin to image the red blood cell surface. Scalebar is 10 μm.

7. Sensor internalization in mammalian cells is minimal when performed on ice.

To prevent internalization of the macromolecules into the mammalian cell interior, the cells were incubated on ice throughout the measurement. We confirmed using confocal microscopy that the majority of the sensors are localized on the cell surface and appear to be bound homogeneously, as shown in Fig. S3.7. In Fig S3.7, the sensors were not added at the same concentration to test for robustness against internalization across a wide range of sensor concentrations. In all of our reported measurements, we did not observe significant internalization of neither the biotin anchor nor the antibody when performing the experiments on ice.

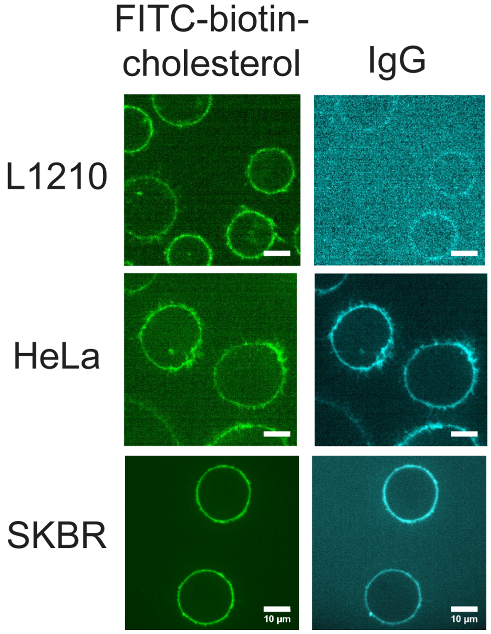

Figure S3.7. Representative confocal images of three cancer cell types incubated with biotin-FITC-cholesterol construct (left) and surface-bound IgG (right). Majority of the signal is localized on the cell surface. This is a test to check for sensor internalization, so these images do not correspond to the same sensor concentrations and the arbitrary image intensity is not reflective of the crowding state of the cells. All scale bars are 10 μm.

8. Bound antibody concentration normalized by the bound biotin-FITC-cholesterol concentration is independent of heterogeneities in cell size.

The advantage of using the DNA-based FITC-biotin-cholesterol sensor is that this construct acts as a proxy of cell size and cell membrane surface area. A plot of normalized bound antibody signal (i.e., antibody intensity divided by the FITC intensity) as a function of forward scattering (a metric of cell size) is shown in Fig. S3.8. We observe a constant normalized bound antibody across a 6x change in forward scattering, which indicates that the variations in cell membrane area is properly accounted for in our calculation of cell heterogeneities. By obtaining the fraction of bound antibodies by dividing against the FITC signal, we are normalizing for cell heterogeneities in shape and surface area. This normalization enables a measurement of population heterogeneities within a sample, which we cannot do using the dextran sensors.

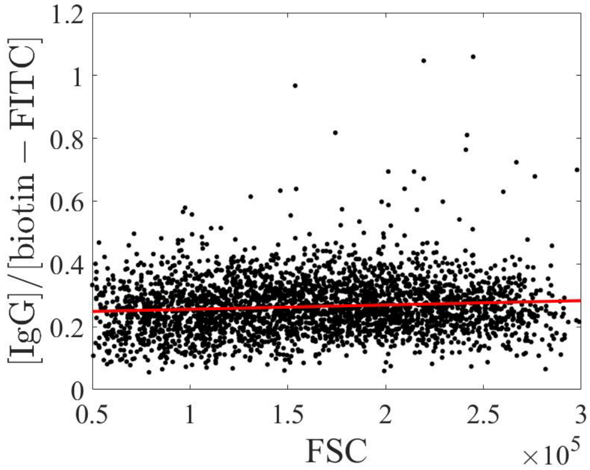

Figure S3.8. Representative flow cytometry data for HeLa cells of normalized antibody signal as a function of forward scatter. Black dots are individual cells and the red line is a linear fit to the data. We obtain approximately zero slope in the linear fit, which indicates that the normalized antibody concentration is independent of cell size.

Chapter IV. Additional supplemental figures

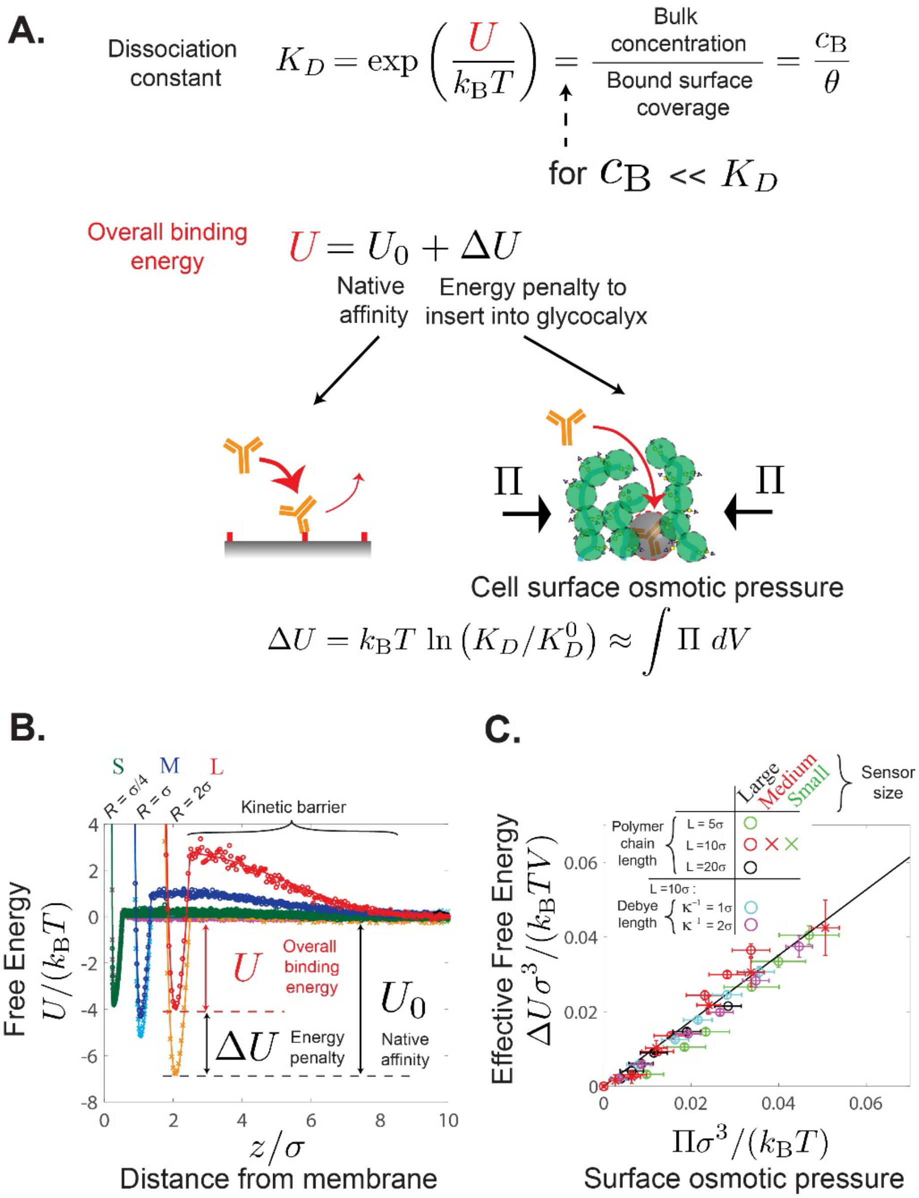

Figure S4.1. Macromolecular crowding on membrane surfaces is described by a surface osmotic pressure.

(A) The dissociation constant of a surface-binding macromolecule is given by its binding energy, U, which is a sum of the intrinsic affinity, U_0, and the penalty due to crowding, ΔU. The energy penalty posed by surface crowding is directly related to the change in normalized binding affinity, K_D/K_D^0, and the osmotic pressure of the surface, Π.

(B) Coarse-grained molecular dynamics (MD) simulations were used to calculate the binding energy curves for spherical sensors of sizes $\sigma_s = 0.25\sigma$ (small), σ (medium), and 2σ (large), where σ is the coarse-grained effective diameter of a glycocalyx polymer chain. The depth of the minimum gives the effective binding energy at equilibrium. The surface contains polymers with contour length 10σ at a concentration of $\sim 10{,}000/\mu m^2$.

(C) The energy penalty across various sensor sizes, surface polymer lengths, and surface polymer charges all collapse onto a unifying scaling line when the data are plotted against the osmotic pressure generated by the surface polymers, Π. The multi-domain proteins on the surface have varying lengths and are either charged (cyan and magenta circles) or neutral (all other symbols). Electrostatic interactions among the polymers are modeled by a Yukawa potential with different Debye lengths, κ^{-1}. The energy penalty ΔU is normalized by the effective volume of the sensor immersed inside the crowded surface, V, and Π is calculated via the Irving-Kirkwood virial stress tensor. Error bar indicates standard deviations of ensembles over all time and particles. The solid line is given by analytical theory and is not a fit.

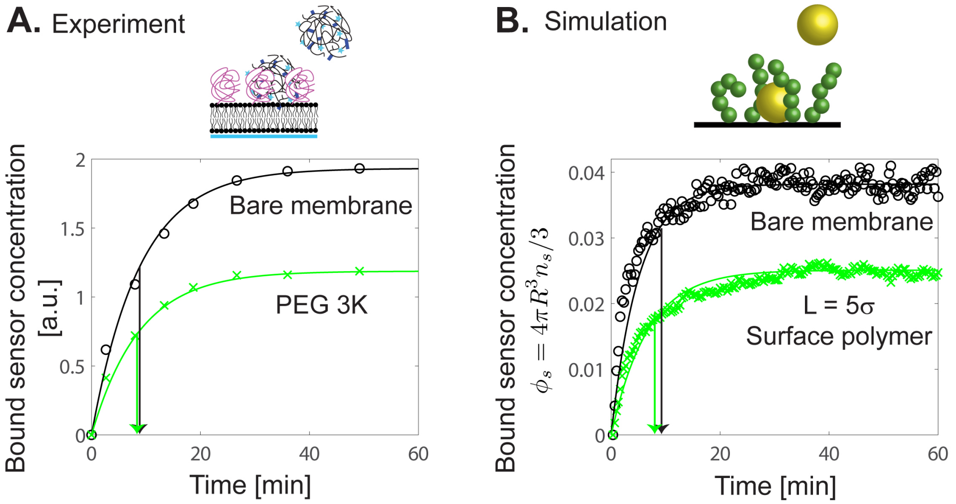

Figure S4.2. Sensor binding on crowded membrane surfaces approaches equilibrium at similar rates for bare and crowded surfaces.

(A) Bound dextran-40k sensor concentration as a function of time on bare membrane beads (black symbols) and crowded PEG 3K surfaces (green symbols) saturates with the same time constant. Solid curves are a fit to a first-order kinetic rate law, C/C_∞ = 1 - exp(-t/τ). The characteristic decay time towards equilibrium is τ = 8.86 min for bare membranes and τ = 8.74 min for crowded PEG 3K surfaces.

(B) MD simulations of sensor binding on bare surfaces (black symbols) and crowded surfaces (green symbols), where $\phi_s = 4\pi R^3 n_s/3$ is the volume fraction of bound sensors at the surface. The characteristic decay time towards equilibrium is τ = 9.8 min for bare membranes and τ = 9.0 min for crowded polymer surfaces.

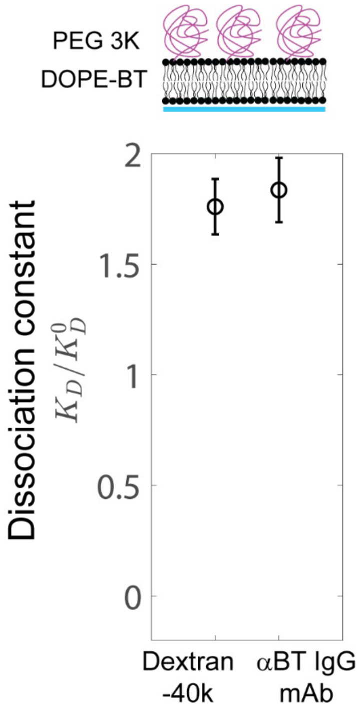

Figure S4.3. The crowding penalties reported by the large dextran-40k sensor and anti-biotin IgG antibodies are quantitatively similar on crowded reconstituted surfaces, consistent with their similar size (~10 nm).

We confirmed that our alternative measurement using antibodies provides a readout of crowding consistent with the dextran-40k sensor.

Experimental materials and resources

Purified human Glycophorin A (CD235a) extracellular domain with a C-terminal 6x-His tag (catalog number: 16018-H08H) was purchased from Sino Biological US Inc.
Alexa Fluor 647 labeled anti-biotin (BK-1/39; catalog number: sc-53179 AF647) was purchased from Santa Cruz Biotechnology. Monoclonal antibodies against human Glycophorin AB (CD235ab) (HIR2; catalog number: 306602) and human CD47 (CC2C6; catalog number: 323102) were purchased from BioLegend.

BD Microtainer Contact Activated Lancet, 30 G x 1.5 mm, low flow, purple (catalog number: 366592) was purchased from BD Biosciences.

Lectins Sambucus Nigra Lectin (SNA; catalog number: L-1300), Fluorescein labeled Agaricus bisporus lectin (ABL; catalog number: FL-1421), Fluorescein labeled Erythrina cristagalli lectin (ECL; catalog number: FL-1141), and Wheat Germ Agglutinin (WGA; catalog number: L-1020-10) were purchased from Vector Laboratories.

1,2-dioleoyl-sn-glycero-3-(N-(5-amino-1-carboxypentyl)iminodiacetic acid)succinyl (DGS-Ni-NTA; catalog number: 709404), 1,2-dioleoyl-sn-glycero-3-phosphocholine (DOPC; catalog number: 850375), 1,2-dioleoyl-sn-glycero-3-phospho-L-serine (DOPS; catalog number: 840035), 1,2-dioleoyl-sn-glycero-3-phosphoethanolamine-N-(cap biotinyl) (Biotinyl Cap PE; catalog number: 870273), 1,2-dioleoyl-sn-glycero-3-phosphoethanolamine-N-methoxy(polyethylene glycol)-2000 (PEG2k PE; catalog number: 880130), 1,2-dioleoyl-sn-glycero-3-phosphoethanolamine-N-methoxy(polyethylene glycol)-3000 (PEG3k PE; catalog number: 880330) were purchased from Avanti polar lipids.

Silica microspheres (4.07µm; catalog code: SS05002; lot number: 12602 and 6.84µm; catalog code: SS06N; lot number: 4907) were purchased from Bangs laboratories. Cholesterol NHS (catalog number: CSL02) was purchased from Nanocs Inc. Cholesterol-PEG-Amine, MW 1k (catalog number: PLS-9961) was purchased from Creative PEGWorks. Dextran, Amino, 10,000 MW (10k dextran; catalog number: D1860), Dextran, Amino, 40,000 MW (40k dextran; catalog number: D1861), Alexa Fluor 647 NHS Ester (Succinimidyl Ester) (NHS-AF647; catalog number: A20006), Alexa Fluor 555 NHS Ester (Succinimidyl Ester) (NHS-AF555; catalog number: A20009), Alexa Fluor 488 NHS Ester (Succinimidyl Ester) (NHS-AF488; catalog number: A20000) , BODIPY FL NHS Ester (Succinimidyl Ester) (NHS-BODIPY; catalog number: D2184) were purchased from Invitrogen.

NHS-Fluorescein (5/6-carboxyfluorescein succinimidyl ester) (NHS-FITC; catalog number: 46409), DyLight 650-4xPEG NHS Ester (catalog number: 62274), EZ-Link Hydrazide-Biotin (hydrazide-biotin; catalog number: 21339), EDC (1-ethyl-3-(3-dimethylaminopropyl)carbodiimide hydrochloride) (catalog number: 77149), Zeba Spin Desalting Column, 7K MWCO (catalog number: 89882) were purchased from Thermo Scientific.

Proteinase K from Tritirachium album (catalog number: P2308) and sialidase from Clostridium perfringens (C. welchii) (catalog number: N2876) were purchased from Sigma. FITC-biotin-DNA-cholesterol construct (5'-FITC-TTTTTT-biotin-TTT-cholesterol-3') was purchased from Integrated DNA Technologies, Inc.

MATLAB educational license was obtained from MathWorks Inc. UC Berkeley's BRC High Performance Computing Savio cluster with NVIDIA K80 GPU were used for molecular simulations. Attune NxT Acoustic Focusing Cytometer (ThermoFisher Scientific) was used for all of the flow cytometer experiments.

References

1 Doi, M. *Soft matter physics*. (Oxford University Press, 2013).

2 Hall, D. & Minton, A. P. Macromolecular crowding: Qualitative and semiquantitative successes, quantitative challenges. *Biochimica et Biophysica Acta (BBA) - Proteins and Proteomics* **1649**, 127-139 (2003).

3 Kim, J. S. & Yethiraj, A. Effect of macromolecular crowding on reaction rates: A computational and theoretical study. *Biophys J* **96**, 1333-1340 (2009).

4 Minton, A. P. Effective hard particle model for the osmotic pressure of highly concentrated binary protein solutions. *Biophys J* **94**, L57-L59 (2008).

5 Stachowiak, J. C., Schmid, E. M., Ryan, C. J., Ann, H. S., Sasaki, D. Y., Sherman, M. B., Geissler, P. L., Fletcher, D. A. & Hayden, C. C. Membrane bending by protein–protein crowding. *Nature Cell Biology* **14**, 944-949 (2012).

6 Rubinstein, M. & Colby, R. H. *Polymer physics*. (OUP Oxford, 2003).

7 Halperin, A. Polymer brushes that resist adsorption of model proteins: Design parameters. *Langmuir* **15**, 2525-2533 (1999).

8 Halperin, A., Fragneto, G., Schollier, A. & Sferrazza, M. Primary versus ternary adsorption of proteins onto peg brushes. *Langmuir* **23**, 10603-10617 (2007).

9 Halperin, A. & Zhulina, E. B. Atomic force microscopy of polymer brushes: Colloidal versus sharp tips. *Langmuir* **26**, 8933-8940 (2010).

10 Louis, A. A., Bolhuis, P. G., Meijer, E. J. & Hansen, J. P. Density profiles and surface tension of polymers near colloidal surfaces. *The Journal of Chemical Physics* **116**, 10547-10556 (2002).

11 Weeks, J. D., Chandler, D. & Andersen, H. C. Role of repulsive forces in determining the equilibrium structure of simple liquids. *Journal of Chemical Physics* **54**, 5237-5247 (1971).

12 Milner, S. T., Witten, T. A. & Cates, M. E. Theory of the grafted polymer brush. *Macromolecules* **21**, 2610-2619 (1988).

13 Son, S., Takatori, S. C., Belardi, B., Podolski, M., Bakalar, M. H. & Fletcher, D. A. Molecular height measurement by cell surface optical profilometry (csop). *Proceedings of the National Academy of Sciences* **117**, 14209-14219 (2020).

14 Kremer, K. & Grest, G. S. Molecular dynamics (md) simulations for polymers. *Journal of Physics: Condensed Matter* **2**, SA295-SA298 (1990).

15 Anderson, J. A., Lorenz, C. D. & Travesset, A. General purpose molecular dynamics simulations fully implemented on graphics processing units. *Journal of Computational Physics* **227**, 5342-5359 (2008).

16 Glaser, J., Nguyen, T. D., Anderson, J. A., Lui, P., Spiga, F., Millan, J. A., Morse, D. C. & Glotzer, S. C. Strong scaling of general-purpose molecular dynamics simulations on gpus. *Computer Physics Communications* **192**, 97-107 (2015).

17 Armstrong, J. K., Wenby, R. B., Meiselman, H. J. & Fisher, T. C. The hydrodynamic radii of macromolecules and their effect on red blood cell aggregation. *Biophys J* **87**, 4259-4270 (2004).

18 Jung, H., Yang, T., Lasagna, M. D., Shi, J., Reinhart, G. D. & Cremer, P. S. Impact of hapten presentation on antibody binding at lipid membrane interfaces. *Biophys J* **94**, 3094-3103 (2008).

19 Yang, T., Baryshnikova, O. K., Mao, H., Holden, M. A. & Cremer, P. S. Investigations of bivalent antibody binding on fluid-supported phospholipid membranes: The effect of hapten density. *Journal of the American Chemical Society* **125**, 4779-4784 (2003).
